# Content-adaptive LSB steganography with saliency fusion, ACO dispersion, and hybrid encryption with ablation study

**DOI:** 10.1038/s41598-025-33920-9

**Published:** 2025-12-29

**Authors:** Ahmed Aljughaiman, Rana Alrawashdeh

**Affiliations:** 1 Department of Computer Networks and Communications, College of Computer Sciences and Information Technology, King Faisal University, 31982 Al-Ahsa, Saudi Arabia; 2 Department of Information and Computer Science, College of Computing and Mathematics, King Fahd University of Petroleum and Minerals, 31261 Dhahran, Saudi Arabia

**Keywords:** ACO, Hybrid encryption, LSB, Sailency map, Steganography, Engineering, Mathematics and computing

## Abstract

Image steganography is a security technique that conceals secret information within digital images in such a way that makes the hidden content imperceptible to human vision and difficult to detect statistically. The main challenge in image steganography lies in achieving an optimal balance among imperceptibility, embedding capacity, and security. To address these limitations, this paper proposes a content-adaptive Least Significant Bit (LSB) steganography framework that integrates saliency-guided embedding, Ant Colony Optimization (ACO)-based dispersion, and hybrid encryption to improve both invisibility and confidentiality. The system embeds secret data in low-sensitivity regions identified by a robust saliency fusion map, minimizing visual distortion. A block-wise ACO mechanism distributes embedding indices spatially across the image to prevent clustering artifacts and enhance undetectability. The framework removes side-information dependency by regenerating embedding indices deterministically from RSA-derived seeds, ensuring reproducibility during extraction. A hybrid cryptographic module combining Advanced Encryption Standard-Galois/Counter Mode (AES-GCM) and Advanced Encryption Standard-Optimal Asymmetric Encryption Padding (RSA-OAEP), with optional Hamming (7,4) error correction, guarantees confidentiality and reliable key recovery. The proposed framework demonstrates near-lossless imperceptibility, achieving PSNR values of 59.7–60.2 dB for 64 × 64 secret images and up to 64.5 dB for 32 × 32 secrets, with SSIM consistently above 0.999 and MSE below 0.07. Under capacity variations with Bits Per Pixel (BPP), the proposed system exhibits a clear rate–distortion behavior as PSNR decreases from 61.23 to 55.17 dB, while SSIM remains above 0.9978. All index-selection modes (ACO, random, perm, and saliency_topk) differ by less than 0.5 dB, confirming the stability of the LSB-robust content map, with saliency_topk achieving the highest PSNR $\approx$ 60.16 dB. From a security perspective, the proposed ACO-guided scheme exhibits random-level detectability against modern CNN-based steganalyzers. Overall, the proposed method offers high imperceptibility, stable and lossless extraction under no-attack conditions, accurate payload rate control, and strong resistance to CNN-based steganalysis.

## Introduction

The information security discipline of steganography enables the covert concealment of communication by embedding secret data into digital media, including audio, video, and images^1,2^. Among these media, digital images are the most widely used carriers because they are ubiquitous and provide a large amount of redundant data suitable for hiding information^3,4^. In image steganography, a secret image (or message) is embedded inside a cover image in such a way that the resulting stego image remains visually indistinguishable from the original, preserving complete or near-complete visual transparency^5,6^. Despite extensive research, the field still faces significant challenges, as steganographic schemes must simultaneously maintain high image quality, provide sufficient embedding capacity, and ensure robustness against both classical and modern Deep Learning (DL)-based steganalysis techniques^7,8^. This trade-off between imperceptibility, capacity, and detectability continues to drive the development of more advanced, content-adaptive, and security-aware steganographic frameworks.

The main goal of image steganography is to hide information within digital images such that the embedded data remains imperceptible to the Human Visual System (HVS) and difficult to detect by statistical tests, while still being reliably recoverable at the receiver side^9,10^. It is challenging to achieve this goal because steganographic schemes must simultaneously satisfy four often conflicting requirements: (i) imperceptibility, typically quantified by high PSNR and SSIM values, low Mean Squared Error (MSE), and the absence of visible artifacts; (ii) capacity, expressed through the payload rate in BPP or bits per non-zero AC coefficient (bpnzAC) in transform-domain methods; (iii) robustness against common image processing operations, such as JPEG compression, rescaling, and noise addition; and (iv) security against modern deep CNN-based steganalysis tools, including Xu-Net, Ye-Net, and SRNet. In practice, improving one of these objectives often degrades another; for example, increasing the payload rate generally makes stego images more susceptible to detection, highlighting an inherent trade-off between capacity, imperceptibility, and detectability^11,12^.

Image steganography underpins a wide spectrum of practical applications across security, healthcare, and digital media domains^9,13^. Typical use cases include: (i) secure covert communication that can evade censorship, traffic monitoring, and protocol-aware inspection; (ii) medical imaging privacy protection, where sensitive patient information or audit trails are embedded within DICOM images without compromising diagnostic fidelity; (iii) copyright protection and digital rights management through invisible forensic watermarks that enable ownership verification and dispute resolution; (iv) content authentication and tamper detection using fragile or semi-fragile marks that signal unauthorized modifications; (v) device provenance tracking and the tagging of IoT telemetry data to support secure logging, accountability, and traceability; and (vi) large-scale media archiving and retrieval, where invisible indexing or retrieval cues are embedded directly into multimedia content to facilitate efficient search and organization^14^. These diverse and increasingly demanding application scenarios further motivate the development of steganographic systems that can jointly balance imperceptibility, embedding capacity, robustness to processing and channel distortions, and resistance to modern steganalysis, in an application-aware and security-conscious manner.

In this work, the proposed framework for LSB steganography uses an LSB-robust content map together with block-wise ACO for index selection to regenerate two separate index sets through RSA-derived seeds, thus eliminating the requirement for storing side information. The system implements AES–GCM encryption with RSA–OAEP key wrapping and optional Hamming (7,4) protection for the wrapped key to provide complete confidentiality and authenticated recovery. The system demonstrates enhanced imperceptibility and reliable recovery under specific attacks because of its strong indexing mechanism and ACO’s spatial distribution pattern while maintaining acceptable execution time and complete reproducibility between different environments. In this work, we will try to answer the following research questions:

1. Does ACO-based index selection outperform random, perm, and saliency_topk at matched payload sizes?
2. Does the LSB-robust content map improve recovery compared to a non-robust map?
3. How does increasing BPP (0.1−0.4) affect imperceptibility and recovery, and where is the knee point?
4. How do embedding/extraction times scale with image size and ACO hyperparameters?
5. Does ACO yield more spatially dispersed embeddings than global top-*K*, and is dispersion tied to better imperceptibility?
6. Which component (LSB zeroing, blur, quantization, ACO) most contributes to robustness and quality?

The goals of our work are to develop a content-adaptive LSB steganography framework-guided by a robust content map and ACO-that ensures confidentiality and authenticity, preserves visual quality, attains stable recovery under common attacks, and remains fully reproducible without storing side information.

1. To achieve better cover–stego quality than random, perm, and saliency_topk at matched payloads; measured by PSNR, SSIM, Image Fidelity (IF), and MSE.
2. To reduce index drift between embedding and extraction via LSB-robust indexing; measured by Jaccard similarity of index sets and Bit Error Rate (BER).
3. To quantify the marginal effect of Hamming (7,4) on key recovery and overall success; measured by key-recovery rate and key-bit BER.
4. To regenerate indices exactly across runs/platforms using RSA-derived, domain-separated seeds; measured by exact index match.

The paper has been organized as follows. The second section covers the related work to this work. The third section covers the proposed methodology. The fourth section discusses the experiment results and analysis, and the fifth section presents the future work, while the sixth section concludes the paper.

## Related work

The protection of Electronic Medical Images (EMIs) stands as a vital matter because of its importance for real-time healthcare operations. LSB- and Most Significant Bit (MSB)-based techniques serve as traditional steganographic methods to embed data within images. Yet, they present restricted Payload Capacity (PC) and visibility problems. Researchers have started to hide information in the Region of No Interest (RONI) to protect essential image regions, especially when working with medical data. Sharma et al.^15^ conducted a comparative analysis between Edge-Based Steganography (EBS) and Block-Based Steganography (BBS), which were applied to the RONI of normalized cover images. The researchers tested their approach using 5856 chest X-ray images to show EBS outperformed BBS in terms of real-time capabilities and computational speed while maintaining high imperceptibility and achieving 100% embedding rates. The authors demonstrated how RONI-based embedding techniques can protect extensive medical data in operational healthcare settings.

The current research in image steganography emphasizes developing methods that combine superior imperceptibility with robustness and high embedding capacity. The widespread adoption of LSB substitution as a method continues because of its basic nature, but it produces noticeable distortions and remains susceptible to statistical steganalysis attacks. Researchers have integrated DL methods, including autoencoders, Generative Adversarial Networks (GANs), and neural decoders, to improve visual quality and minimize distortion in stego images. Verma et al.^16^ presented a hybrid system that merged LSB embedding with DL-based image decoding and SSIM perceptual loss functions to produce better visual quality and less pixel degradation. The research demonstrated how combining image enhancement techniques with decoder-based recovery methods produces high-quality imperceptible steganography, especially when visual integrity matters in specific applications.

Wang^17^ presented AttnEdge as an advanced edge detection system that unites Pixel Difference Convolution (PDC) with self-attention mechanisms to detect global dependencies while enhancing feature representation. The model includes post-processing operations that combine Gaussian blur with adaptive thresholding to enhance edge continuity and reduce noise. AttnEdge outperforms both Canny and PiDiNet models by producing superior SSIM and PSNR results on BSDS500 dataset, which indicates better structural accuracy and robustness.

The current research in image steganography aims to enhance the balance between embedding capacity and visual imperceptibility and resistance to steganalysis. The LSB substitution method provides simple computation and high capacity, but remains susceptible to statistical and visual attacks. The introduction of transform-domain techniques, including Discrete Wavelet Transform (DWT) and Discrete Cosine Transform (DCT), alongside hybrid models that combine encryption with error correction and DL methods, has improved robustness. Jasim and Kurnaz^18^ developed an advanced steganographic system through the combination of ACO with the LSB method. The ACO-LSB method used pheromone-guided exploration to select optimal embedding positions, which enhances security and maintains image quality. The ACO-LSB performs data embedding through decoy bits for integrity verification while implementing checksum validation to detect tampering. Their proposed method demonstrated improved embedding capacity by 30% through experimental testing on the BOSSbase dataset while achieving imperceptibility with 40.5 dB PSNR and 0.98 SSIM. Their proposed method decreases detection rates by 15% compared to Particle Swarm Optimization (PSO)-based methods, which results in high resistance to steganalysis.

The hybrid image steganography system of Younus and Hussain^4^ used cryptographic compression together with an advanced embedding technique to achieve security features, PC, and image imperceptibility. The preprocessing stage of this method contains two phases, which first apply the Vigenère cipher to encrypt the secret message, followed by Huffman coding compression to reduce message size and boost payload efficiency. The actual process implements an enhanced Exploiting Modification Direction (EMD) method to embed encoded digits into the cover image by performing small modifications to pixel values. The evaluation results demonstrated that this method outperformed traditional EMD and Optimized Exploiting Modification Direction (Opt-EMD) and LSB-based schemes by achieving PSNR values above 55 dB and SSIM scores of approximately 0.99, and PCs reaching 52,400 bytes without degrading image quality. The stego-images maintained statistical distributions that were nearly identical to their original counterparts based on chi-square steganalysis tests.

Zhang et al.^19^ proposed a new adaptive steganographic algorithm to solve the problems caused by lossy image processing in Online Social Networks (OSNs), including JPEG recompression and enhancement filtering. The Minimizing Channel Error Rate (MINICER) technique divides channel errors into two independent categories: steganography-related and steganography-independent components. Their proposed method differs from previous methods as it embeds data into channel-processed covers and then replaces the corresponding original cover components to create the stego. The method preserves robustness while maintaining undetectability. The authors conducted extensive experiments using both simulated and real-world platforms (e.g., Facebook) to show that their method outperforms Generalized Minimum-Error Adaptive Steganography (GMAS) and Joint Channel Rate and Image Scaling Steganography (JCRIS) in terms of message recovery, accuracy, and steganalysis resistance.

Subramanian et al.^3^ performed a comprehensive review of image steganography progress by organizing methods into three categories: traditional, CNN-based, and GAN-based approaches. Traditional methods, including LSB and Pixel Value Differencing (PVD) that offer basic security but provide minimal protection against attacks. The authors present CNN-based models (U-Net, Xu-Net) and GAN-based frameworks (CycleGAN, SteganoGAN) that achieved better imperceptibility and steganalysis resistance through their reported PSNR values that reach up to 64.7 dB. The authors emphasized the need to develop hybrid models and employ advanced metrics and datasets to build more secure and efficient steganographic systems.

The researchers in^20^ have recently addressed the trade-off between embedding capacity and imperceptibility in image steganography by combining cross-diagonal embedding PVD with the Modulus Function (MF) technique, guided by edge area patterns. This method aims to simultaneously enhance embedding efficiency and visual quality without compromising security. This approach was evaluated on 14 public datasets and achieved an average embedding capacity of 3.18 BPP, while maintaining high imperceptibility, with PSNR values exceeding 40 dB and SSIM scores above 0.98. The use of edge-aware block pattern embedding also contributed significantly to preserving visual fidelity. In addition to improved performance metrics, the method demonstrated robustness against Regular Singular (RS) steganalysis attacks, highlighting its strength in maintaining security under statistical detection.

The evaluation of classical steganographic techniques has been performed to determine how embedding capacity interacts with imperceptibility and robustness. Lu et al.^21^ evaluated three spatial domain methods: LSB, MSB, and PVD in detail across grayscale and color images. The authors tested the methods using PSNR, MSE, and SSIM tests on benchmark cover images, including Lena, Baboon, and Peppers. The PSNR and SSIM values were highest for LSB, indicating superior visual quality with minimal distortion, and PVD provided higher embedding capacity. The MSB technique provided better data security than LSB but resulted in more distortion.

The current research in robust steganography emphasizes error reduction in lossy environments, such as OSNs. MINICER represents a new framework developed by Zeng et al.^22^, which addressed both steganography-related and independent distortion sources by embedding messages in channel-processed covers and strategically replacing elements in the original image to maintain security and robustness. The method removes non-steganographic distortions (e.g., JPEG recompression, enhancement filtering) and uses the wet paper coding model to protect unstable elements from modification. The experimental results showed better performance in terms of message recovery and detection resistance than JCRIS and GMAS in both simulated and real-world platforms. MINICER achieved robustness through its design, which eliminates the requirement for error correction codes while preserving high imperceptibility and low error rates during aggressive image post-processing.

Research on DL methods, including GANs for improving image steganography, security, and adaptability, has grown substantially throughout the last few years. The embedding algorithms S-UNIWARD, HILL, and MiPOD operate with fixed cost functions that depend on local image structures. Yet, these limitations restrict their adaptability and resistance to steganalysis attacks. Wang et al.^23^ created a GAN-based framework that trains adaptive embedding cost maps through a dual-stream U-Net generator that integrates Convolutional Spatial Attention (CSA) with image enhancement modules. The Automatic Steganographic Distortion Learning framework with GAN (ASDL-GAN) and UT-GAN models received enhancements through adversarial training and architectural improvements for cost learning. The approach encountered difficulties during training stability and detection precision. The research of Steganographic Pixel-wise Actions and Rewards with Reinforcement Learning (SPAR-RL) and CF-UT-GAN explored RL and feedback-based optimization to enhance embedding decisions. The approach delivers improved cost map accuracy through its detailed attention to edge and texture regions. The experimental results demonstrated that their method enhances resistance to advanced steganalysis models, which resulted in a significant advancement for adaptive image steganography with attention guidance.

In this context, Beggari et al.^24^ introduced a robust and imperceptible dual-stage watermarking framework specifically designed for telemedicine applications. Their method first embeds sensitive textual information into a grayscale patient image using a DCT-K-means scheme to identify optimal embedding regions. Next, integrates the resulting watermarked image into a medical color image through a DWT Self-Organizing Map (SOM) approach. To further enhance data integrity, Reed-Solomon (RS) coding is applied to correct transmission errors. Experimental results showed that this hybrid system achieved high imperceptibility PSNR $\approx$ 45 dB and strong robustness Normalized Cross-Correlation (NCC) $\approx$ 0.99 against various attacks, outperforming several state-of-the-art watermarking methods. Their work highlighted the growing trend toward combining transform-domain techniques with intelligent clustering and error-correction mechanisms to achieve secure and resilient medical image watermarking.

Said et al.^25^ proposed a frequency-domain watermarking framework for medical image protection based on the Fractional Discrete Cosine Transform (FDCT), Mellin transform, and Schur decomposition. Their method was evaluated using three core watermarking metrics: capacity, imperceptibility, and robustness. In terms of imperceptibility, their proposed approach achieved an average PSNR of 39.38 dB and an excellent SSIM of 0.9998, indicating almost perfect structural similarity between the original and watermarked images. Multi-dataset experiments were conducted on MRI, X-ray, fundus, and CT/MRI images to confirm further that high visual quality is preserved across diverse modalities. Regarding capacity, the method embeds 2592 Bits Per Image (BPI), corresponding to 0.07031 BPP. This embedding rate outperforms several FDCT-based schemes, yet remains moderate compared with high-capacity watermarking methods designed for richer payloads. Robustness is assessed under a wide range of attacks, including average, Gaussian, median filtering, JPEG compression at different quality factors, histogram equalization, rotation, cropping, scaling, Gaussian noise, salt-and-pepper noise, and speckle noise. The reported NCC values frequently exceed 0.95, and reach around 0.98 for common operations, such as filtering and JPEG compression, Q = 30–90, demonstrating strong resilience suitable for telemedicine workflows. However, the scheme exhibits limitations under certain conditions. The performance degrades in the presence of speckle noise, where NCC drops to about 0.90, and under more severe geometric transformations (rotation and scaling), where robustness becomes weaker relative to some competing techniques. In addition, the triple-transform pipeline (FDCT-Mellin-Schur) introduced non-negligible computational overhead: for a 192$\times$192 image, embedding and extraction times are on the order of one second, increasing further for higher resolutions. These factors may restrict real-time deployment and highlight the need for future work on geometric-invariant embedding strategies and reduced computational complexity while preserving the favorable trade-off between imperceptibility, capacity, and robustness.

Similarly, Beggari et al.^26^ proposed a robust medical image watermarking framework that integrates the Ridgelet transform, QR decomposition, Ant Colony Optimization (ACO), and adaptive Quantization Index Modulation (QIM) to enhance telemedicine security. Their method was extensively evaluated using three main categories of metrics: capacity, imperceptibility, and robustness. In terms of capacity, the scheme achieves a high payload of 73728 BPI, corresponding to 1.125 BPP, which is sufficient to embed detailed patient metadata and a SHA-256 authentication hash without compromising diagnostic utility. Regarding imperceptibility, quantitative results show an average PSNR of 48.63 dB and SSIM of 0.9917, indicating that the visual quality of the watermarked medical images remains very close to the original, even in sensitive regions, such as tumor boundaries. The robustness of their proposed approach was assessed under a wide range of image processing attacks, including additive noise, filtering, histogram equalization, and JPEG compression. The authors reported Normalized Correlation (NC) values that exceed 0.97 for most non-geometric attacks, and remain around 0.9623 for JPEG compression with quality factor Q = 30, demonstrating strong resilience in typical telemedicine workflows. However, the scheme still exhibits notable limitations. First, robustness degrades under severe geometric distortions: large rotations $(75^\circ )$ and cropping 25% reduce NC to approximately 0.75 and 0.82, respectively, due to subband misalignment in the transform domain. Second, the combination of multi-scale Ridgelet processing and iterative ACO optimization introduces non-negligible computational overhead, with average embedding and extraction times of 0.89 s and 0.52 s per image, which may hinder strict real-time applications. These limitations motivate future work on geometric-invariant features and lighter optimization strategies to maintain the favorable trade-off between capacity, imperceptibility, and robustness.

The current state of image steganography shows multiple operational limitations, which prevent its widespread adoption. The current methods fail to achieve proper equilibrium between capacity, imperceptibility, and robustness when dealing with high payload amounts. The current techniques lack protection against statistical and DL-based steganalysis attacks, which require new methods for secure data embedding. The current approaches for image steganography primarily work with fixed-size images and basic datasets, yet researchers have not adequately studied their performance with different image types and resolutions. The absence of complete evaluation metrics prevents scientists from performing objective assessments, as these metrics must measure both visual quality through PSNR and SSIM and attack resistance. The implementation of complex or adaptive steganographic schemes faces two main challenges, which are achieving real-time operation and maintaining efficient computation. The existing research gaps create opportunities to create methods that combine high capacity with security features, general applicability, and efficient computation to connect theoretical concepts with real-world usage.

## Methodology

**Fig. 1:**
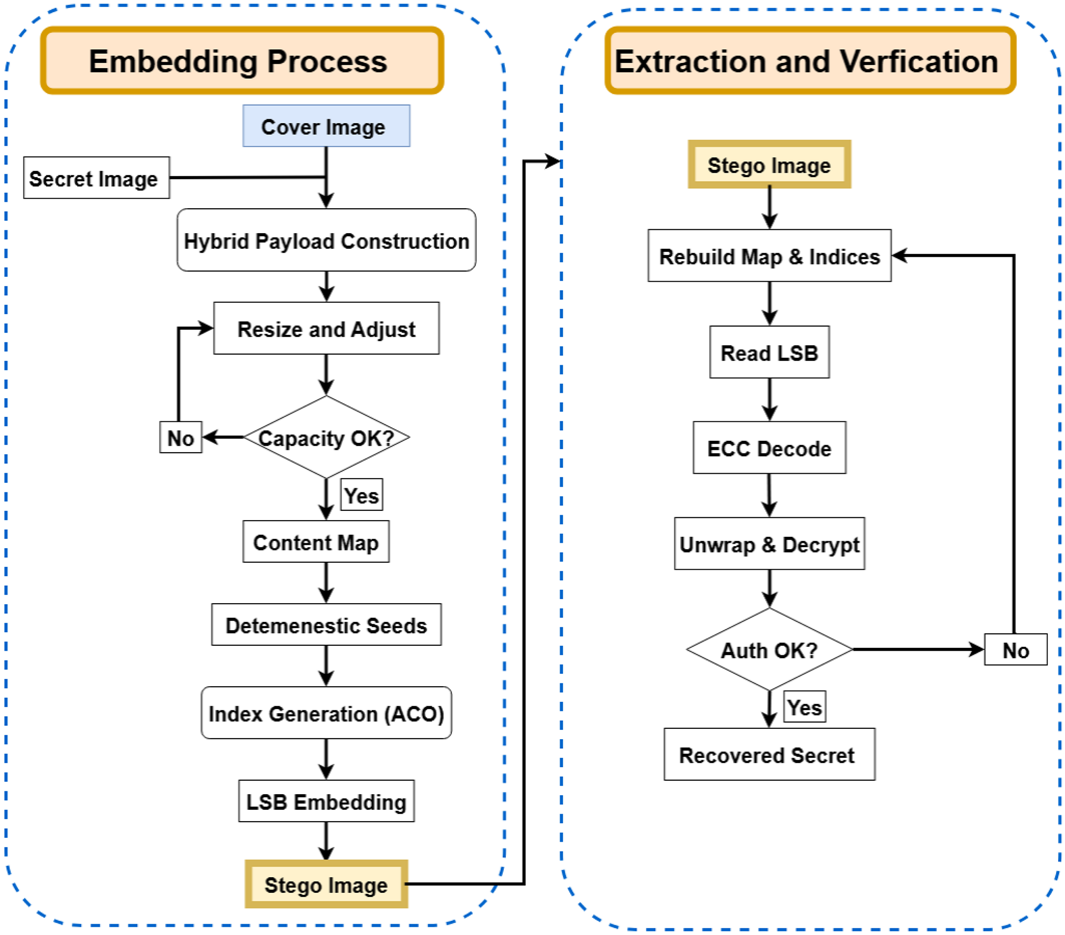
The proposed approach.

The proposed methodology, as shown in Fig. 1 follows a structured workflow that begins with preparing the input images and ends with securely extracting the hidden secret. First, both the cover image and the secret image are converted into 8-bit grayscale format to ensure consistency. If a specific embedding rate (BPP) is required, the size of the secret image is automatically adjusted so that the payload fits within the available capacity of the cover image. Next, a hybrid encryption process is applied to protect the secret: the secret is encrypted using AES-GCM to produce the ciphertext, nonce, and authentication tag, while the AES key itself is wrapped using RSA-OAEP. An optional Hamming(7,4) error-correcting code may also be added to improve robustness against bit errors. All encrypted components are then combined into a single compact bitstream ready for embedding. To determine where embedding should occur, a robust content map is generated from the cover image. This map is stabilized by removing LSB noise, applying a light Gaussian blur, and combining both saliency and gradient-magnitude cues, which are then uniformly quantized into discrete levels. Using this content map, two deterministic seeds are derived from the receiver’s RSA public key-one for embedding the wrapped key and another for the AES-GCM data. These seeds ensure that the same embedding positions can be regenerated during extraction without storing any side information. An ACO algorithm is then applied over non-overlapping $8 \times 8$ blocks of the image to select embedding locations that are spatially well distributed and lie in visually complex regions, thereby reducing noticeable distortion. During the embedding stage, the LSB of each selected pixel is first cleared and then replaced with the corresponding payload bit, producing the final stego image.

In the extraction stage, the system rebuilds the same content map, regenerates the same index sets, and reads back the hidden bits from the LSBs of the indexed pixels. If enabled, the Hamming(7,4) decoder corrects single-bit errors in the wrapped key. The AES key is then recovered using RSA-OAEP and used in AES-GCM decryption of the ciphertext. The recovered secret is accepted only if the authentication tag is verified successfully, which guarantees data integrity and authenticity. Together, these steps provide a content-adaptive, cryptographically secure, and reproducible steganography framework with reliable recovery and high visual quality.

### Dataset acquisition

This research performs testing operations on two independent datasets throughout this study. The USC-SIPI image dataset functions as the secret dataset, as it contains digitized images^27^. This database contains organized volumes that sort images based on their characteristics. The images within each volume present different pixel dimensions, which range from 256$\times$256 pixels to 512$\times$512 pixels and 1024$\times$1024 pixels.

The USC-SIPI dataset contains synthetic and structured images with controlled edge and texture properties, which makes it appropriate for testing edge-based embedding methods. The method demonstrates strong resistance to various image types and environmental conditions because the dataset includes multiple image variations. The BOSSbase dataset contains 10,000 grayscale images, which measure 512$\times$512 pixels and exist in Portable Gray Map (PGM) format^28^. The images were captured by seven different cameras before JPG format conversion. The BOSSbase dataset version 1.01 represents the current version of the collection. The researchers chose BOSSbase and USC-SIPI datasets as they present essential image characteristics for steganography applications. The high-resolution natural grayscale images in BOSSbase help evaluate real-world imperceptibility.

### Data preparation and preprocessing

The input preparation stage ensures that both the cover and secret images are brought into a consistent and capacity-aware format before any embedding takes place. First, the cover image is loaded and converted into an 8-bit grayscale representation, with its resolution standardized so that subsequent processing operates on uniform image dimensions. The secret image is then loaded and similarly converted to 8-bit grayscale. When the system operates under a specified embedding rate, expressed in BPP, the size of the secret image is automatically adjusted to match the available capacity of the cover image. This capacity is computed as follows:

1 $$\begin{aligned} C_{\text {bits}} = \left\lfloor bHW \right\rfloor \end{aligned}$$

where $b$ denotes the target BPP, and $H$ and $W$ are the height and width of the cover image, respectively. The secret image is resized or synthesized so that its total bit length, together with the required cryptographic overhead (e.g., wrapped keys, nonces, and authentication tags), fits within $C_{\text {bits}}$ without overflow. In this way, the input preparation step guarantees that the steganographic channel is neither underutilized nor overloaded, and that the subsequent embedding process can proceed in a controlled and reproducible manner. If the secret image exceeds the allowable embedding capacity determined by the target BPP and the dimensions of the cover image, it is reduced through a controlled resizing process. This reduction typically employs interpolation methods, such as bilinear or bicubic scaling, producing a smaller secret image whose bitstream fits within the capacity constraint.

Although this shrinking operation inevitably removes some fine details, it preserves the essential visual content–including the overall structure, dominant patterns, and semantic information-which is sufficient for steganographic purposes. The objective is not to reproduce the original secret image pixel-for-pixel, but rather to ensure that the extracted version remains recognizable and meaningful while avoiding payload overflow and maintaining high imperceptibility in the cover image.

### Payload construction (Hybrid Crypto with Optional Elliptic Curve Cryptography (ECC))

The payload construction stage is responsible for securely transforming the secret data into a cryptographically protected bitstream suitable for embedding. First, an AES-128 session key is generated to provide efficient and authenticated symmetric encryption. The secret image (or message) is then encrypted using AES-GCM, producing three essential components: a nonce for initialization, a ciphertext representing the encrypted data, and an authentication tag that enables integrity verification during extraction. To ensure that only the intended receiver can recover the AES session key, the key is wrapped using RSA-OAEP with a 2048-bit public key, providing strong asymmetric protection.

To enhance robustness against potential bit errors during embedding or extraction, an optional Hamming(7,4) error-correcting code may be applied to the wrapped key. This lightweight ECC ensures correct recovery of the AES key even when minor LSB disturbances occur. Finally, all components are concatenated into a single payload bitstream in the following structured form:

2 $$\begin{aligned} P = \text {wrapped\_key}(\pm \text {ECC}) \; || \; \text {nonce} \; || \; \text {ciphertext} \; || \; \text {tag} \end{aligned}$$

where $||$ denotes bitwise concatenation. This compact and ordered construction ensures confidentiality, integrity, and reliable recovery of the secret during the extraction phase, while remaining compatible with pixel-based embedding constraints.

### Robust content map for index selection (LSB-robust)

The construction of the content map represents a critical stage in ensuring that the embedding process is both visually imperceptible and reproducible. To achieve this, the cover image undergoes several preprocessing operations designed to stabilize the low-level pixel structure and produce a reliable measure of embedding suitability. First, the LSBs of the cover image are zeroed to remove self-induced noise and ensure that the later indexing process is not influenced by random LSB variations. A light Gaussian blur with a $3 \times 3$ kernel is then applied to further smooth local fluctuations and enhance the stability of subsequent feature extraction. Next, two complementary feature maps are computed: a spectral-residual saliency map, which highlights visually surprising or attention-grabbing regions, and a gradient magnitude map, which captures structural edges and texture-rich areas. These two maps are fused multiplicatively to form a unified content map as shown below:

3 $$\begin{aligned} F = \text {Saliency} \times \text {Gradient} \end{aligned}$$

which emphasizes pixels that are both visually complex and structurally significant–ideal locations for embedding modifications that remain imperceptible to the human eye.

To ensure consistency between embedding and extraction, the fused map is uniformly quantized into 512 discrete levels. This quantization step provides a stable ranking of pixels based on their embedding suitability and guarantees that the same ordered index list can be regenerated deterministically during extraction. By integrating saliency, edge information, and quantization, the resulting LSB-robust content map forms the foundation for adaptive, reproducible, and minimally intrusive data embedding.

Zeroing all LSB values is a deliberate trade-off that removes one visually insignificant BPP in order to gain a much more stable and reproducible embedding process. The LSB plane contributes minimally to perceived image quality, so its modification produces changes that are typically imperceptible to the HVS. At the same time, clearing LSBs suppresses small random fluctuations (sensor noise, compression artifacts, platform-dependent rounding) that would otherwise perturb saliency and gradient responses, causing index drift between embedding and extraction. Thus, this quantization step slightly alters the original image but is justified because it ensures a deterministic, LSB-robust content map and reliable re-generation of embedding indices without storing side information. Because setting the LSB to zero removes only 1 bit of tiny random fluctuation, but the remaining 7 BPP still contain all the meaningful structure (edges, textures, regions). Gaussian blur operates on those remaining bits and on spatial relationships between pixels, so its output is still highly meaningful.

### Deterministic index generation

The deterministic index generation stage ensures that both the embedding and extraction processes rely on exactly the same pixel locations without storing or transmitting any side information. This design enhances security, reduces metadata leakage, and guarantees reproducibility across platforms. To achieve this, the system derives two independent seeds directly from the receiver’s RSA public key. Each seed is produced by hashing the public key concatenated with a domain-separating constant, allowing the system to isolate the indexing of different payload components. Specifically, the seeds are computed as:

4 $$\begin{aligned} \text {seed}_{\text {key}} = \text {SHA256}(\text {pub} \, || \, 0x00) \end{aligned}$$

5 $$\begin{aligned} \text {seed}_{\text {gcm}} = \text {SHA256}(\text {pub} \, || \, 0x01) \end{aligned}$$

where $||$ denotes byte-level concatenation. These domain-separated seeds ensure that the positions used to embed the wrapped AES key are generated independently from those used for the AES-GCM record, even though both are derived from the same public key.

Using the fused and quantized content map, the system then invokes a deterministic selection routine to generate two disjoint index sets: one for the key material $K_{\text {key}}$ and one for the encrypted data $K_{\text {gcm}}$. The selection process guarantees that no pixel is used for both segments, ensuring:

6 $$\begin{aligned} K_{\text {key}} \; \cap \; K_{\text {gcm}} = \emptyset \end{aligned}$$

By combining deterministic seed generation with the stable ranking provided by the LSB-robust content map, the system can regenerate the exact same embedding indices during extraction without ever storing or transmitting them. This eliminates the need for side information and strengthens both the security and reproducibility of the steganographic framework.

### ACO-based index selection ant colony optimization (ACO)

The ACO-based index selection stage refines the process of choosing embedding locations by leveraging the exploratory behavior of ACO to achieve spatial dispersion and minimize visible artifacts. To begin, the cover image is partitioned into non-overlapping $8 \times 8$ blocks, providing a structured representation that aligns with localized texture and complexity patterns. Each block is then assigned an initial pheromone level, forming the basis for probabilistic decision-making during the optimization process.

Next, a heuristic value is computed for every block by averaging the content strength derived from the fused map $F$. Blocks with higher saliency–gradient responses naturally receive higher heuristic scores, indicating their suitability for embedding in visually complex regions. During the ACO iterations, artificial ants traverse the blocks based on a probability model driven by pheromone intensity and heuristic strength. The parameters $\alpha = 1$, $\beta = 2$, and evaporation rate $\rho = 0.2$ govern the balance between exploration and exploitation. With each iteration, pheromone trails are reinforced on blocks selected by higher-performing ants, while less favorable trails gradually evaporate.

From each chosen block, up to 48 of the most suitable pixels are selected according to their local ordering in the content map. This results in a spatially diverse set of candidate locations that avoids clustering artifacts commonly associated with simpler index-generation techniques. The process is executed twice: once to generate the index set $K_{\text {key}}$ for the wrapped AES key, and again to generate $K_{\text {gcm}}$ for the AES-GCM record. By ensuring that these two sets remain disjoint and widely distributed, the ACO-based strategy enhances imperceptibility, improves robustness against localized distortions, and contributes to the overall quality and security of the embedding procedure.

### Embedding procedure

The embedding procedure performs the actual hiding of the cryptographically protected payload within the cover image. Before any modification takes place, the system verifies that the total payload length does not exceed the available capacity defined by the selected index sets (e.g., $K_{\text {key}}$ and $K_{\text {gcm}}$). This check ensures that all bits of the constructed payload $P$ can be embedded without overflow or truncation. The cover image is then treated in vectorized form, denoted by $x$, so that pixel updates can be applied using index-based operations.

For each selected pixel position, the LSB is first cleared to remove any existing information in that bit plane. This is achieved by applying a bitwise AND with the decimal value 254 (binary = 11111110), as follows:

7 $$\begin{aligned} x[i] = x[i] \wedge 254 \end{aligned}$$

where $x[i]$ is the intensity value at pixel index $i$, and $\wedge$ denotes the bitwise AND operation. After clearing the LSB, the system sequentially embeds the payload bits. For a given payload bit $P_j$ and its corresponding embedding location $K[j]$, the update rule is:

8 $$\begin{aligned} x[K[j]] = \bigl (x[K[j]] \wedge 254\bigr ) \; \vee \; P_j \end{aligned}$$

where $\vee$ denotes the bitwise OR operation. This operation preserves the higher-order bits of the pixel while replacing the LSB with the desired payload bit.

Once all bits of the wrapped key and AES-GCM record have been embedded over their respective index sets, the modified vector $x$ is reshaped back into its original two-dimensional image form. The resulting stego image $\hat{I}$ is obtained via the following formula:

9 $$\begin{aligned} \hat{I} = \operatorname {unvec}(x) \end{aligned}$$

where $\operatorname {unvec}(\cdot )$ denotes the inverse of the vectorization operation. In this way, the embedding procedure produces a visually similar stego image that contains the full encrypted payload while introducing only minimal perturbations to the cover. In theory, the true embedding capacity of an image varies with its content–texture-rich regions can hide more modifications, while smooth regions are far more sensitive to distortion. However, the BPP metric used in most steganography research is defined as a normalized payload rate, rather than a measure of the image’s semantic or perceptual embedding capacity.

### Extraction and verification

The extraction and verification stage mirrors the embedding workflow to ensure that the hidden payload is recovered accurately and securely. Upon receiving the stego image, the system begins by reconstructing the same LSB-robust content map $\hat{F}$ that was used during embedding. This reconstruction applies the identical preprocessing pipeline–including LSB zeroing, Gaussian smoothing, saliency estimation, gradient computation, and quantization–to guarantee that the content map remains stable even if minor LSB disturbances have occurred. Using $\hat{F}$ and the previously defined deterministic seed derivations, the system regenerates the exact same index sets for the wrapped key and AES-GCM data, ensuring perfect alignment without requiring any stored side information.

Once the index sets have been regenerated, the system reads the LSBs from the corresponding pixel locations to recover the embedded bitstreams. This yields two distinct outputs: (i) the wrapped AES key (optionally encoded with Hamming(7,4)), and (ii) the AES-GCM record composed of the nonce, ciphertext, and authentication tag. If error correction was enabled during embedding, a Hamming(7,4) decoder is applied to correct any single-bit errors in the wrapped key, improving reliability under mild distortions.

Following extraction, the wrapped key is unwrapped using RSA-OAEP with the receiver’s private key, yielding the AES-128 session key. This session key is then used to decrypt the ciphertext via AES-GCM. As part of the authenticated decryption process, the GCM authentication tag is verified to ensure both data integrity and authenticity. The secret image (or message) is released only if the authentication tag is valid; otherwise, the recovery process is rejected. Through this detailed sequence of reconstruction, deterministic indexing, bitstream extraction, cryptographic unwrapping, and integrity verification, the system guarantees secure, reproducible, and trustworthy recovery of the embedded secret.

### Ablation protocol and reproducibility

The ablation protocol evaluates the proposed steganographic framework under ten controlled experimental configurations to measure its robustness, consistency, and reproducibility. Each setting varies along four dimensions: (i) the index selection mode (ACO, random, perm, saliency_topk); (ii) the error-correction setting (Hamming(7,4) enabled or disabled), (iii) the payload regime (fixed secret image sizes of $64\times 64$ or $32\times 32$ pixels, or variable capacity constrained by $\textrm{BPP}\in \{0.1,\,0.2,\,0.4\}$), (iv) and the attack condition (no attack vs. JPEG compression at $Q=75$). For each configuration, two complementary result tables are produced: Part A reports imperceptibility metrics (PSNR, SSIM, IF, and MSE) comparing the cover and stego images, while Part B evaluates recovery performance, including secret-image PSNR, embedding and extraction times, payload utilization, and extraction success.

To ensure experimental fairness, all images are processed in grayscale, runtime is measured using perf_counter, and robust indexing consistently applies LSB removal, a $3\times 3$ Gaussian blur, and 512-level uniform quantization. The ACO parameters are fixed across all runs with block size 8, 8 ants, 10 iterations, $\alpha =1$, $\beta =2$, evaporation rate $\rho =0.2$, and up to 48 top pixels harvested per block.

A key feature of the protocol is its deterministic re-indexing strategy: during extraction, the exact index sets used for embedding are regenerated from SHA-256 domain-separated seeds derived from the RSA public key, removing the need for any stored metadata and guaranteeing run-to-run consistency. Finally, the stability rationale confirms that the combination of LSB zeroing, light Gaussian smoothing, and uniform quantization stabilizes the pixel ranking process, prevents index drift between embedding and extraction, and preserves high image quality (PSNR and SSIM) across all experimental variants.

## Results and analysis

The evaluation of the proposed method relies on four essential criteria, which include imperceptibility, capacity, security, and robustness. The evaluation of imperceptibility depends on five metrics, which include MSE, Root Mean Squared Error (RMSE), PSNR, SSIM, and IF. The system reports two capacity metrics, which include PC in bits and BPP. The security evaluation depends on two CNN-based steganalyzers, which are Xu-Net and Ye-Net. The evaluation of robustness includes testing the system under typical disturbances, such as JPEG compression and noise addition, when possible. The following section presents a detailed description of all evaluation metrics together with experimental parameters.

### Imperceptibility

**MSE:** is used to represent the average of the MSE between the pixels of the cover image and the stego image. Here, the MSE value is calculated by using Eq. 10. The range of MSE value is between 0 and 1, and the lower values (that are close to zero) are better than the higher values (that are closer to one)^23,29^. MSE can be calculated as follows^22,30^:

10 $$\begin{aligned} \text {MSE} = \frac{1}{mn} \sum _{i=1}^{m} \sum _{j=1}^{n} (I(i,j) - K(i,j))^2 \end{aligned}$$

where m, n: Image dimensions, I(i,j): Pixel value of the original image, K(i,j): Pixel value of the stego image

**PSNR:** is used to describe the quality of the stego-image based on the Human Visual System (HVS) with its normal value as 30 dB^5,6^. When the PSNR value is greater than 30 dB, it means that the data inserted in the image is invisible to the human eye^7,8^. The PSNR value can be calculated by Eq. 11, and the higher values of PSNR are better than the lower values. PSNR can be calculated as follows^11,12^:

11 $$\begin{aligned} \text {PSNR} = 10 \times \log _{10} \left( \frac{{\text {MAX}}^2}{{\text {MSE}}} \right) \end{aligned}$$

where MAX: Maximum possible pixel value, MSE: Mean Squared Error between original and stego image

**SSIM:** is used to measure the similarity between the cover image and the stego image. The result of the SSIM value remains between 0 and 1. When the SSIM value is close to 1, this means that the stego image is similar to the cover image and is of high quality. The SSIM value is calculated using Eq. 12^14^.

12 $$\begin{aligned} \text {SSIM}(I, K) = \frac{{(2\mu _I\mu _K + C_1)(2\sigma _{IK} + C_2)}}{{(\mu _I^2 + \mu _K^2 + C_1)(\sigma _I^2 + \sigma _K^2 + C_2)}} \end{aligned}$$

where:

$$\begin{aligned}&I, K: \text {Two different images} \\&\mu _I, \mu _K: \text {Mean intensity of images } I \text { and } K \\&\sigma _I, \sigma _K: \text {Standard deviation of intensities of images } I \text { and } K \\&\sigma _{IK}: \text {Covariance of intensities of images } I \text { and } K \\&C_1, C_2: \text {Constants to stabilize the division} \end{aligned}$$

**IF:** is another metric to identify the quantity of errors in pixel values. If the value is closer to 1, it means that the two images are more similar^31^. The IF value is calculated using Eq. 13^32,33^.

13 $$\begin{aligned} \text {IF}(I, K) = \frac{1}{N} \sum _{i=1}^{N} \left( 1 - \frac{|I_i - K_i|}{\max (I_i, K_i)}\right) \end{aligned}$$

where:

$$\begin{aligned}&\text {IF}(I, K): \text {Information Fidelity between images } I \text { and } K \\&N: \text {Total number of pixels in the images} \\&I_i, K_i: \text {Pixel values at position } i \text { in images } I \text { and } K \end{aligned}$$

### Capacity

**PC:** is a measure of how much data can be hidden in the cover image by the proposed steganography, and it will be quite dependent on the size of the cover image^34^. Equation 14 shows the calculation of this measure^35,36^.

14 $$\begin{aligned} \text {PC} = {{\text {T} \times N \times 8}} \end{aligned}$$

where T: Total pixels of cover image, N: bytes per pixels: 1 for grayscale or 3 for color image

**BPP:** is the average value of the embedding per pixel of the whole image^29,30^. BPP can be calculated by Eq. 15^37^.

15 $$\begin{aligned} \text {BPP} = \frac{{\text {PC (bits)}}}{N} \end{aligned}$$

where PC (bits): Total payload capacity in bits, N: Total number of pixels in the image

**Bit Error Rate (BER):** measures the proportion of incorrectly extracted bits to the total number of embedded bits^31,34^.

16 $$\begin{aligned} \text {BER} = \frac{N_{\text {error}}}{N_{\text {total}}} \end{aligned}$$

**Embedding time vs Extraction time:** The process of converting a cover image into a stego image through payload bit insertion defines the embedding time, while the extraction time measures the duration needed to extract embedded bits from a stego image. The computational cost of the system increases when either stage reaches higher values of embedding time, as it requires more powerful transforms, error correction methods, larger data payloads, and multiple optimization loops, which decrease system performance and prevent real-time operation. The pipeline operates at higher efficiency when extraction times are low, which results in better throughput, lower energy consumption, and simpler deployment on limited devices.

The embedding time calculation uses the following formula:

17 $$\begin{aligned} T_{\textrm{emb}} \;=\; t_{\textrm{end}}^{(E)} \;-\; t_{\textrm{start}}^{(E)} \end{aligned}$$

The extraction time calculation follows this formula:

18 $$\begin{aligned} T_{\textrm{ext}} \;=\; t_{\textrm{end}}^{(X)} \;-\; t_{\textrm{start}}^{(X)} \end{aligned}$$

The ten settings in Table 1 demonstrate uniform high cover-stego fidelity through $\textrm{SSIM}\ge 0.9990$ and $\textrm{IF}\ge 0.99997$ and $\textrm{PSNR}$ values close to $60\,\textrm{dB}$ for a $64{\times }64$ secret at fixed capacity. The saliency_topk method produces the highest imperceptibility results at $(64{\times }64,\ \text {no attack})$ with $\textrm{PSNR}=60.162\,\textrm{dB}$ and $\textrm{MSE}\approx 0.06265$ while ACO and random and perm follow with slightly lower values of $\approx 59.86\,\textrm{dB}$ and $\approx 59.69\,\textrm{dB}$ and $\approx 59.70\,\textrm{dB}$ respectively. The distortion values between index methods show minimal difference as they vary by less than $0.5\,\textrm{dB}$ at this specific payload level. The image quality remains unaffected when ECC is enabled or disabled because ECC modifies the payload distribution instead of affecting coefficient positions. The image quality improves significantly when reducing the secret size from $64{\times }64$ to $32{\times }32$ according to ACO with $\textrm{PSNR}=64.500\,\textrm{dB}$ and $\textrm{SSIM}=0.999927$ and $\textrm{MSE}=0.02307$. The embedding perturbation dominates the perceptual measures because the cover-stego metrics show no significant difference between the JPEG flag and the no-attack case according to ACO with $59.664$ vs. $59.714\,\textrm{dB}$. The BPP-constrained runs demonstrate the expected relationship between BPP and image quality because $\textrm{BPP}_{\text {cfg}}$ increases from 0.1 to 0.2 to 0.4, the PSNR values decrease from 61.226 to 58.217 to 55.171 dB, and the MSE values increase from 0.04903 to 0.09803 to 0.19769 while SSIM remains above 0.9978 at BPPcfg = 0.4. The results demonstrate that higher payloads produce a direct relationship with decreased image quality based on the specified rate.

**Table 1:** Cover vs stego quality using secret image size 64$\times$64.

#	Index mode	ECC	Size	BPP_cfg	Attack	PSNR (C vs S)	SSIM (C vs S)	IF (C vs S)	MSE (C vs S)
1	ACO	True	(64, 64)	–	none	59.714	0.999526	0.999990	0.069458
2	ACO	False	(64, 64)	–	none	59.863	0.999547	0.999991	0.067104
3	random	True	(64, 64)	–	none	59.686	0.999130	0.999990	0.069897
4	perm	True	(64, 64)	–	none	59.702	0.999061	0.999990	0.069649
5	saliency_topk	True	(64, 64)	–	none	60.162	0.999549	0.999991	0.062649
6	ACO	True	(32, 32)	–	none	64.500	0.999927	0.999997	0.023071
7	ACO	True	(64, 64)	–	jpeg	59.664	0.999517	0.999990	0.070248
8	ACO	True	–	0.1	none	61.226	0.999803	0.999993	0.049030
9	ACO	True	–	0.2	none	58.217	0.999146	0.999986	0.098030
10	ACO	True	–	0.4	none	55.171	0.997848	0.999972	0.197685

C vs S: cover vs stego

The second part of Table 2 presents ten different configurations from Part A by showing capacity control results and runtime measurements and recovery outcomes. The pipeline performs lossless extraction in 8 out of 10 runs under benign conditions because $\textrm{PSNR}_{\text {Sec vs Ex}}=\infty$ and Recovered = Yes. The system operates at a BPP range of 0.139 to 0.134 (rows 1 through 4), which results in 36.6 kbits of payload data on a 262144-pixel canvas. When using a small secret in row 6, the system achieves 0.0458 BPP (12 kbits) with complete recovery. The system maintains precise rate control because it achieves target BPP values of 0.097 and 0.195 and 0.397 for b values of 0.1, 0.2, and 0.4 while utilizing 99% of the available budget (rows 8-10). The system achieves lossless extraction in all cases. The system requires different amounts of time for embedding and extraction operations, which range from 0.10 to 15.22 seconds and 0.10 to 8.95 seconds, respectively. The time required for both embedding and extraction operations grows longer when the target BPP increases from 0.1 to 0.2 and then to 0.4. The system fails to recover data when JPEG compression reaches Q=75 (row 7), which demonstrates its non-robust nature against compression attacks. The system fails to recover data in row 5 due to its sensitivity to index/selection parameters, although it maintains high fidelity in Part A. The table demonstrates both exact rate control and complete lossless data retrieval during no-attack scenarios, yet execution duration depends on selection choices and payload sizes, and JPEG robustness needs further development.

**Table 2:** Cover vs stego capacity using secret image size 64 × 64.

#	BPP_cfg	Attack	PSNR (S vs X)	Embed-t	Extra-t	BPP_used	Payload	Payload used	Recovered
1	–	None	$\infty$	15.217086	7.28159	0.139526	262144	36576	✓
2	–	None	$\infty$	7.740237	6.93117	0.133667	262144	35040	✓
3	–	None	$\infty$	0.157694	0.177501	0.139526	262144	36576	✓
4	–	None	$\infty$	0.376473	0.364362	0.139526	262144	36576	✓
5	–	None	–	0.10022	0.099883	0.139526	262144	36576	✗
6	–	None	$\infty$	6.691847	5.418414	0.045776	262144	12000	✓
7	–	Jpeg	–	7.118804	7.956097	0.139526	262144	36576	✗
8	0.1	None	$\infty$	6.496444	7.641	0.097046	26214	25440	✓
9	0.2	None	$\infty$	7.621054	8.213185	0.195465	52428	51240	✓
10	0.4	None	$\infty$	10.927019	8.950895	0.397339	104857	104160	✓

Rows (1–10) mirror the configurations in Table 1, enabling one-to-one comparison.

PSNR (S vs X): secret vs extracted secret, Embed-t (s), Extra-t(s), Payload (bits), Payload used (bits)

The cover-stego fidelity remains high across all configurations in Table 3 as SSIM stays above 0.9991, IF reaches 0.99999, and PSNR maintains values near 60 dB when using a 64x64 secret image at fixed capacity. The saliency_topk index mode produces the highest imperceptibility results through its 60.154 dB PSNR value and its lowest MSE measurement, although ACO and random and perm produce results that differ by less than 0.5 dB. The selection of the index does not significantly affect the image quality at this payload level. The activation or deactivation of ECC produces no noticeable impact on image quality because it modifies data distribution patterns instead of affecting coefficient positions. The image quality improves significantly when reducing the secret size to 32x32 pixels because PSNR reaches 64.542 dB, SSIM reaches 0.999928, and MSE reaches 0.022850. The Part A metrics show no significant change when JPEG compression is applied during the ”Attack” phase because the embedding noise affects perceptual quality more than the compression process. The BPP-constrained runs demonstrate the typical relationship between rate and distortion because increasing the $\textrm{BPP}_{cfg}$ value from 0.1 to 0.4 results in decreased PSNR values (61.196 to 58.236 to 55.165 dB) and increased MSE values (0.049370 to 0.097599 to 0.197983) while SSIM values stay above 0.9978. The visual quality depends more on payload rate than index mode selection according to these results.

**Table 3:** Cover vs stego quality using secret image size 128 × 128.

#	Index mode	ECC	Size	BPP_cfg	Attack	PSNR (C vs S)	SSIM (C vs S)	IF (C vs S)	MSE (C vs S)
1	ACO	True	(64, 64)	–	None	59.666	0.999513	0.999990	0.070225
2	ACO	False	(64, 64)	–	None	59.888	0.999543	0.999991	0.066719
3	random	True	(64, 64)	–	None	59.688	0.999122	0.999990	0.069862
4	perm	True	(64, 64)	–	None	59.724	0.999067	0.999990	0.069286
5	saliency_topk	True	(64, 64)	–	None	60.154	0.999547	0.999991	0.062763
6	ACO	True	(32, 32)	–	None	64.542	0.999928	0.999997	0.022850
7	ACO	True	(64, 64)	–	Jpeg	59.692	0.999520	0.999990	0.069801
8	ACO	True	–	0.1	None	61.196	0.999799	0.999993	0.049370
9	ACO	True	–	0.2	None	58.236	0.999154	0.999986	0.097599
10	ACO	True	–	0.4	None	55.165	0.997822	0.999972	0.197983

C vs S: cover vs stego

The configurations in Part B of Table 4 follow the same pattern as Part A to show capacity control, runtime, and recovery results. The pipeline demonstrates perfect bit recovery in 8 out of 10 tested configurations, as it achieves infinite PSNR between the original and extracted images and successfully recovers the data. The system operates at a BPP usage range of 0.134 to 0.140 without BPP constraints while handling 36.6 kbits of data on a 262144-pixel image area. The system operates at 0.0458 BPP, which equals 12 kbits when using the smaller-secret configuration, yet maintains complete lossless recovery. The system maintains precise rate control because it achieves target BPP values of 0.097, 0.195, and 0.397 while using 97–99% of the allocated budget for payload transmission and achieves complete data recovery. The system execution time depends on the specific configuration because embedding operations take between 0.10 and 10.86 seconds, and extraction operations take between 0.10 and 9.05 seconds. The system requires longer processing times for higher target rates because it needs to perform additional write and read operations, but it takes multiple seconds to complete low-rate operations because of map construction and index selection costs. The system demonstrates two instances of failure, which show its sensitivity to specific conditions because JPEG compression at Q=75 prevents recovery and one no-attack scenario results in failure. The table demonstrates that the system maintains exact rate control and achieves complete lossless data recovery during normal operations, while processing time depends on selection and payload parameters, and JPEG resistance needs further development.

**Table 4:** Cover vs stego capacity using secret image size 128 × 128.

#	BPP_cfg	Attack	PSNR (S vs X)	Embed-t	Extra-t	BPP_used	Payload	Payload used	Recovered
1	–	None	$\infty$	12.067302	8.746229	0.139526	262144	36576	✓
2	–	None	$\infty$	6.187544	8.512021	0.133667	262144	35040	✓
3	–	None	$\infty$	0.155935	0.177369	0.139526	262144	36576	✓
4	–	None	$\infty$	0.369213	0.365092	0.139526	262144	36576	✓
5	–	None	–	0.099162	0.097759	0.139526	262144	36576	✗
6	–	None	$\infty$	5.722079	6.959136	0.045776	262144	12000	✓
7	–	Jpeg	–	6.317516	8.705144	0.139526	262144	36576	✗
8	0.1	None	$\infty$	5.952915	8.158916	0.097046	26214	25440	✓
9	0.2	None	$\infty$	6.749431	9.049732	0.195465	52428	51240	✓
10	0.4	None	$\infty$	10.857936	8.560357	0.397339	104857	104160	✓

PSNR S vs X: secret vs extracted secret, Embed-t (s), Extra-t(s), Payload (bits), Payload used (bits)

Rows (1–10) mirror the configurations in Table 3, enabling one-to-one comparison.

The Table 5 (cover size $512{\times }512$) shows that cover-stego fidelity remains consistently high at $\textrm{SSIM}\ge 0.9991$ and $\textrm{IF}\approx 0.99999$ across all configurations while $\textrm{PSNR}$ values cluster at 60$\,\textrm{dB}$ for a 64$\times$64 secret at fixed capacity. The saliency_topk method produces the highest imperceptibility results with $\textrm{PSNR}=60.157\,\textrm{dB}$ and $\textrm{MSE}=0.062717$ while ACO, random, and perm show similar results at 59.68-59.92$\,\textrm{dB}$ because index selection produces minimal changes at this payload level. The change in ECC does not affect the results because it modifies the payload structure instead of altering the placement of coefficients. The reduction of the secret size to 32$\times$32 results in better fidelity with $\textrm{PSNR}=64.567\,\textrm{dB}$ and $\textrm{SSIM}=0.999928$ and $\textrm{MSE}=0.022720$. The embedding rate reduction explains this outcome. The Part A metrics show no significant change when JPEG compression is applied in the ”Attack” field because the embedding perturbations dominate the perceptual measures under this specific setup. The $\textrm{BPP}$-constrained runs demonstrate the expected relationship between rate and distortion because $\textrm{PSNR}$ decreases from 61.262 to 58.222 to 55.166$\,\textrm{dB}$ while $\textrm{MSE}$ increases by a factor of two at each step from 0.048630 to 0.097919 to 0.197926 and $\textrm{SSIM}$ remains above 0.9978. The visual quality depends more on payload rate than index strategy at this resolution.

**Table 5:** Cover vs stego quality using secret image size 512 × 512.

#	Index mode	ECC	Size	BPP_cfg	Attack	PSNR (C vs S)	SSIM (C vs S)	IF (C vs S)	MSE (C vs S)
1	ACO	True	(64, 64)	–	None	59.686	0.999520	0.999990	0.069901
2	ACO	False	(64, 64)	–	None	59.920	0.999549	0.999991	0.066227
3	random	True	(64, 64)	–	None	59.683	0.999120	0.999990	0.069954
4	perm	True	(64, 64)	–	None	59.707	0.999062	0.999990	0.069557
5	saliency_topk	True	(64, 64)	–	None	60.157	0.999552	0.999991	0.062717
6	ACO	True	(32, 32)	–	None	64.567	0.999928	0.999997	0.022720
7	ACO	True	(64, 64)	–	Jpeg	59.697	0.999513	0.999990	0.069721
8	ACO	True	–	0.1	None	61.262	0.999804	0.999993	0.048630
9	ACO	True	–	0.2	None	58.222	0.999138	0.999986	0.097919
10	ACO	True	–	0.4	None	55.166	0.997822	0.999972	0.197926

C vs S: Cover vs Stego

The results of Part B in Table 6 present the same experimental conditions as Part A to show capacity control, runtime, and recovery performance for the 512x512 cover image. The pipeline produces perfect extraction results in 8 out of 10 test cases under benign conditions because the PSNR between the extracted and original data reaches infinity and the recovery process succeeds. The system operates at bit-perfect extraction rates when it does not enforce a BPP limit because it achieves $\textrm{BPP}_{used}$ values between 0.134 and 0.140 while processing 36.6 kbits of data across 262144 pixels. The smaller-secret configuration enables perfect recovery at a $\textrm{BPP}_{used}$ rate of 0.0458, which results in 12 kbits of payload. The system maintains precise rate control because it achieves target rates with high accuracy at 0.097, 0.195, and 0.397 while using 97-99% of the available bits for extraction. The execution duration of the system depends on the selected configuration because embedding operations take between 0.16 and 10.65 seconds, while extraction operations take between 0.18 and 11.30 seconds. The fastest execution times occur when using content-agnostic index modes such as random and perm because higher target BPPs lead to longer processing times for embedding and extraction. The system fails to recover data when JPEG compression reaches Q=75 and when using no attack configuration with specific indexing choices. The results show that the system maintains exact rate control and performs lossless data extraction during normal operations, while processing time depends on selection methods and data volume, and JPEG compression remains a challenge for improvement.

**Table 6:** Cover vs stego capacity using secret image size 512 × 512.

#	BPP_cfg	Attack	PSNR (S vs X)	Embed-t	Extra-t	BPP_used	Payload	Payload used	Recovered
1	–	None	$\infty$	13.483250	6.439786	0.139526	262144	36576	✓
2	–	None	$\infty$	8.243059	6.175495	0.133667	262144	35040	✓
3	–	None	$\infty$	0.259661	0.275765	0.139526	262144	36576	✓
4	–	None	$\infty$	0.593038	0.606186	0.139526	262144	36576	✓
5	–	None	–	0.159626	0.187999	0.139526	262144	36576	✗
6	–	None	$\infty$	5.846836	5.549757	0.045776	262144	12000	✓
7	–	Jpeg	–	8.570792	6.419071	0.139526	262144	36576	✗
8	0.1	None	$\infty$	8.146326	5.866124	0.097046	26214	25440	✓
9	0.2	None	$\infty$	9.069988	6.852381	0.195465	52428	51240	✓
10	0.4	None	$\infty$	10.645520	11.302322	0.397339	104857	104160	✓

PSNR S vs X: Secret vs Extracted secret, Embed-t (s), Extra-t(s), Payload (bits), Payload used (bits)

Rows (1–10) mirror the configurations in Table 5, enabling one-to-one comparison.

**Fig. 2:**
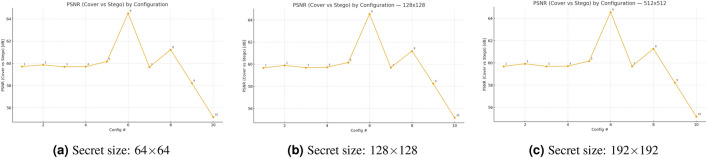
Effect of secret size and configuration on embedding distortion: PSNR (cover–stego) across ten configurations.

The Fig. 2 shows PSNR values between cover images and stego images for ten different embedding configurations at three secret-image sizes (64 × 64, 128 × 128, 192 × 192). The ordering of the curves remains consistent between different sizes because the selection of configuration determines how much distortion occurs during embedding. The PSNR value of configuration #6 reaches approximately 64.5 dB because it uses the lowest effective embedding load, but configuration #10 (BPP-cfg = 0.4) produces the lowest PSNR value of 55.2 dB, which demonstrates the relationship between capacity and distortion. The fixed-payload settings from #1 to #5 show that the content-aware saliency-topk method (#5) produces better results than ACO/random/perm methods with PSNR values of 60.15 dB compared to 59.7–59.9 dB. The selection of indexes through guided methods provides better results than random selection methods. The disabling of ECC in configuration #2 results in a 0.2 dB improvement compared to configuration #1, which uses ECC. The PSNR values for the JPEG case (#7) match those of its non-attack counterpart because PSNR measures embedding distortion rather than channel degradations, yet attack effects appear more in recovery metrics. The PSNR values of all configurations exceed 55 dB, which means the steganographic artifacts remain imperceptible to the human eye, while configuration #6 provides the best results for moderate capacity requirements.

**Fig. 3:**
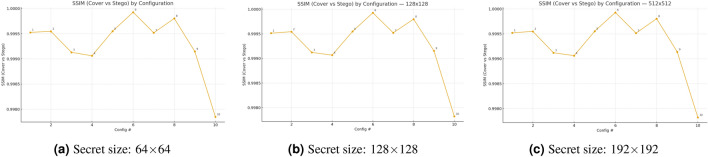
Effect of secret size and configuration on structural fidelity: SSIM (cover–stego) across ten configurations.

The Fig. 3 shows SSIM values between cover images and their stego counterparts for ten embedding configurations at three secret-image dimensions $(64\times 64,\,128\times 128, 512\times 512)$. The three curves maintain the same order of performance at all secret sizes because the selection of configuration determines structural distortion more than the size of the secret. The SSIM value of configuration #6 reaches approximately $0.99993$ because it embeds data with minimal distortion, while configuration #10 with $\textrm{BPP}_{\textrm{cfg}}=0.4$ produces the lowest SSIM value of $0.9978$, which demonstrates the trade-off between capacity and distortion. The fixed-payload configurations from #1 to #5 show that the saliency-topk method outperforms ACO, random, and perm, while disabling ECC in #2 results in a slightly better SSIM than ECC-enabled #1. The JPEG case in #7 shows similar results to its non-attack counterpart because SSIM measures embedding distortion rather than channel degradation effects. The recovery metrics show channel degradations better than SSIM does. All values exceed $0.9978$, which indicates excellent structural fidelity and high imperceptibility, with a minimal difference between the best and worst results of $0.0021$.

**Fig. 4:**
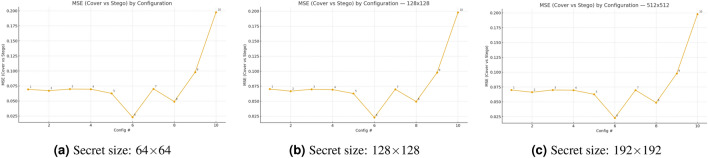
Effect of secret size and configuration on embedding distortion (MSE, cover–stego).

The evaluation of MSE between cover and stego images occurs for ten embedding configurations at three different secret-image sizes as shown in Fig. 4. The ordering of the curves remains identical at all sizes because the selection of configuration produces more impact on distortion than the size of the secret image. The MSE values of configuration #6 remain the lowest at 0.0227-0.0231, while configuration #10 with BPP=0.4 produces the highest MSE at 0.1979, which represents an 8.6-8.7 times increase between the two values. The content-aware saliency-topk method in fixed-payload settings (#1-#5) produces slightly better results than ACO, random, and perm methods, but disabling ECC in #2 results in a minimal improvement over ECC-enabled #1. The MSE values increase steadily when the payload amount grows from #8 to #9 and then to #10.

**Fig. 5:**
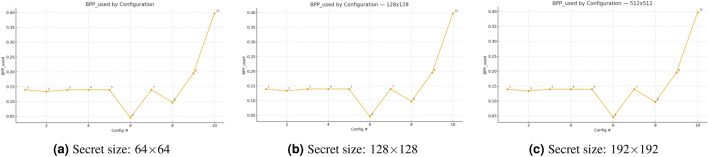
BPP used by configuration across secret sizes: Capacity consumed per pixel for ten configurations.

The Fig. 5 demonstrates the actual payload usage per pixel $\textrm{BPP}_{\text {used}}$ across ten embedding configurations at three different secret-image dimensions $(64\times 64,\, 128\times 128,\, 192\times 192)$. The three curves match each other exactly because the secret image size does not affect the capacity usage, which depends on the configuration settings. The minimum $\textrm{BPP}_{\text {used}}$ value of $0.0458$ appears in configuration #6, while the maximum value of $0.3973$ occurs in configuration #10, representing an $8.7\times$ increase in capacity usage. The results show a direct relationship between $\textrm{PSNR}/\textrm{MSE}$ and the capacity–distortion trade-off. The fixed-payload cluster contains values around $0.1395\,\textrm{bpp}$ for configurations #1 through #5 and #7. The value of $\textrm{BPP}_{\text {used}}$ for the ECC-disabled configuration #2 reaches $0.1337$, whereas the ECC-enabled configuration #1 reaches $0.1395\,\textrm{bpp}$. The target-payload settings follow a direct relationship with their specified values: #8 at $0.1\,\textrm{bpp}$ yields $0.0970$, #9 at $0.2\,\textrm{bpp}$ yields $0.1955$, and #10 at $0.4\,\textrm{bpp}$ yields $0.3973$. The $\textrm{BPP}_{used}$ measurement in the JPEG case (#7) shows the same results as non-attack scenarios because it evaluates embedding load rather than channel-degradation effects.

**Fig. 6:**
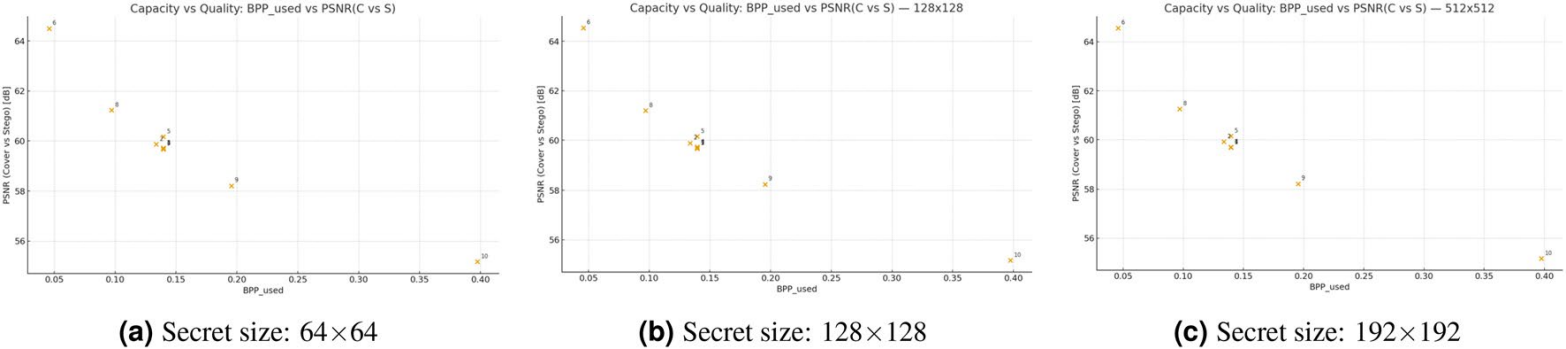
Capacity–quality trade-off across secret sizes: PSNR (cover–stego) versus BPP-used for ten embedding configurations.

Figure 6 presents scatter plots for ten embedding configurations evaluated at three secret-image sizes $(64\times 64,\, 128\times 128,\, 192\times 192)$. The points exhibit a direct relationship between the used embedding rate $\textrm{BPP}_{used}$ and stego quality (PSNR in dB), indicating that configuration choice governs distortion more strongly than secret size. The best quality is achieved by configuration #6 under the lowest load $\left( \textrm{BPP}_{\text {used}}\approx 0.046\right)$ with $\textrm{PSNR}\approx 64.5 \textrm{dB}$, whereas configuration #10, at $\textrm{BPP}_{\text {used}}\approx 0.397$, yields the poorest quality $\left( \textrm{PSNR}\approx 55~\textrm{dB}\right)$. These findings demonstrate how the capacity–distortion trade-off shapes system performance. The fixed-payload settings (#1–#5 and #7) maintain a consistent $\textrm{BPP}_{\text {used}}\approx 0.139$; within this cluster, #5 outperforms the others, and disabling ECC in #2 provides a slight improvement over the ECC-enabled #1.

**Fig. 7:**
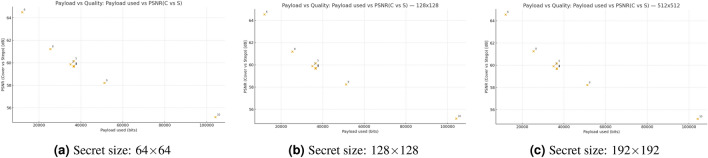
Payload–quality trade-off of the proposed steganographic scheme for different secret sizes.

Figure 7 shows the three scatter plots present the payload-quality trade-off of the proposed steganographic method for secret sizes measuring 64x64 pixels, 128x128 pixels, and 192x192 pixels. The PSNR in dB values show an opposite trend in all panels because higher payload usage leads to reduced stego-image quality. The PSNR values for matched payloads show that smaller secrets produce better image quality than larger secrets as the distortion penalty grows with secret size. The panels show tight clustering patterns, which indicate consistent behavior between different cover-image and secret-image pairs, but the content-specific variations lead to minor scatter. The figure demonstrates the expected relationship between capacity and distortion while showing that decreasing secret size leads to better visual quality at fixed embedding rates.

**Fig. 8:**
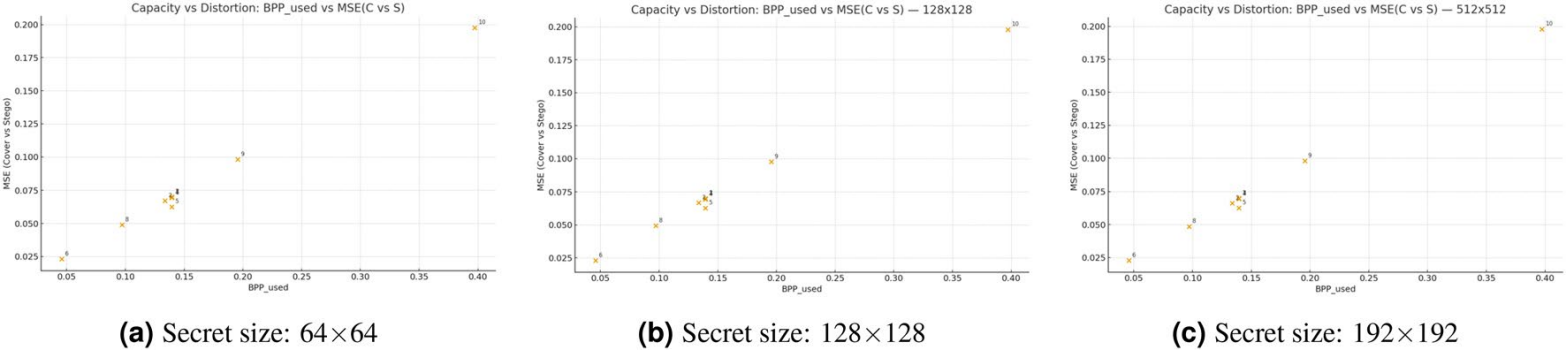
Capacity–distortion trade-off across secret sizes.

Figure 8 shows that the three scatter plots demonstrate how the proposed steganographic scheme handles capacity versus distortion relationships. The horizontal axis displays the used embedding rate $\textrm{BPP}_{used}$ (BPP) and the vertical axis shows the distortion level through the MSE between cover and stego images $\textrm{MSE}(C,S)$. The data points in all three panels (i) $64\times 64$, (ii) $128\times 128$, and (iii) $192\times 192$ demonstrate a direct relationship between $\textrm{MSE}(C,S)$ and $\textrm{BPP}_{used}$ because the distortion increases when the embedding rate grows. The visual quality of the steganographic image decreases when the secret message size increases, as the smaller secret size produces images with lower distortion than the larger ones. The point clouds maintain a small range of dispersion throughout the covers but show increased spread when the embedding rates become higher, which indicates stable performance with occasional content-related variations at extreme points. The proposed method enables users to adjust both embedding rate and secret size for effective distortion management.

## Security analysis

This research assesses security against data-driven steganalysis through the evaluation of two prominent CNN models, Xu-Net and Ye-Net. The spatial domain-based networks use high-pass residual images of grayscale data to train discriminative features, which help detect stego content from cover images. The Xu-Net operates as an efficient detection system because it uses early residual preprocessing and shallow to moderate depth convolutional blocks with batch normalization and truncated or clipped activation functions to detect local embedding artifacts. The Ye-Net operates as a heavier CNN model, which uses high-pass filters similar to Spatial Rich Model(SRM) and deep convolutional and pooling layers to process spatial information for better pattern detection. The analysis includes both point estimates of confusion matrices and complete ROC curves to evaluate threshold-independent separation and detect decision bias that occurs when all images are classified as either cover or stego during distribution changes. The training process of Xu-Net and Ye-Net starts from scratch without pretraining, as we use content-matched pairs of Bossbase images (each cover image has its corresponding stego version) with 160 training pairs and 20 validation and test pairs that result from a deterministic split. In this section, we test and analyze the tradeoff between the payload and security at different BPP values (0.1, 0.2, and 0.4).

### Xu-Net analysis

**Fig. 9:**
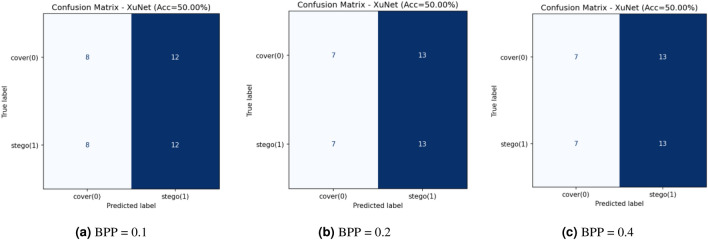
Xu-Net Confusion Using ACO at different BPP.

The confusion matrices of Xu-Net at three different payload rates (BPP = 0.1, 0.2, 0.4) appear in Fig. 9. The overall accuracy reaches 50% in all scenarios, which demonstrates that the model performs Gaussian random at each level. The error distribution shows equal weight in both directions of the panels, while it slightly favors the stego class predictions (12-13 predicted-stego counts compared to 7-8 predicted-cover counts). By the class, the performance results in unhelpful metrics because the detection accuracy reaches 0.60-0.65 for True Positives (TPs) and 0.35-0.40 for True Negatives (TNs), which makes the Matthews correlation equal approximately 0 and macro-F1 to be approximately 0.5. The confusion structure of Xu-Net remains constant between 0.1 and 0.4 BPP as the ACO-based embedding produces stego artifacts that remain unlearnable for this payload range, and Xu-Net achieves chance-level performance without task-specific retraining. To validate our results, we compare our work with the research in^10^ that shows the experimental results with Xu-Net demonstrate the proposed steganographic scheme is quite difficult to detect, even for a strong CNN-based steganalyzer. Across the three datasets (BossBase, Tiny ImageNet, and USC-SIPI) and the two edge operators (Canny and Sobel), Xu-Net’s detection accuracy stays in a relatively low band, roughly between 0.52 and 0.67. Only on the USC-SIPI dataset with Sobel edges does the accuracy reach around 0.67; in most other cases, it is close to 0.52–0.55, which is barely above random guessing (0.50). This means that, although Xu-Net is able to pick up some weak artifacts in specific settings, it cannot reliably distinguish cover from stego images generated by the proposed method. When we compare our work and the above paper, we note that our approach outperforms the above paper in terms of PSNR and SSIM, which increases the imperceptibility while preserving the security at the same time.

**Fig. 10:**
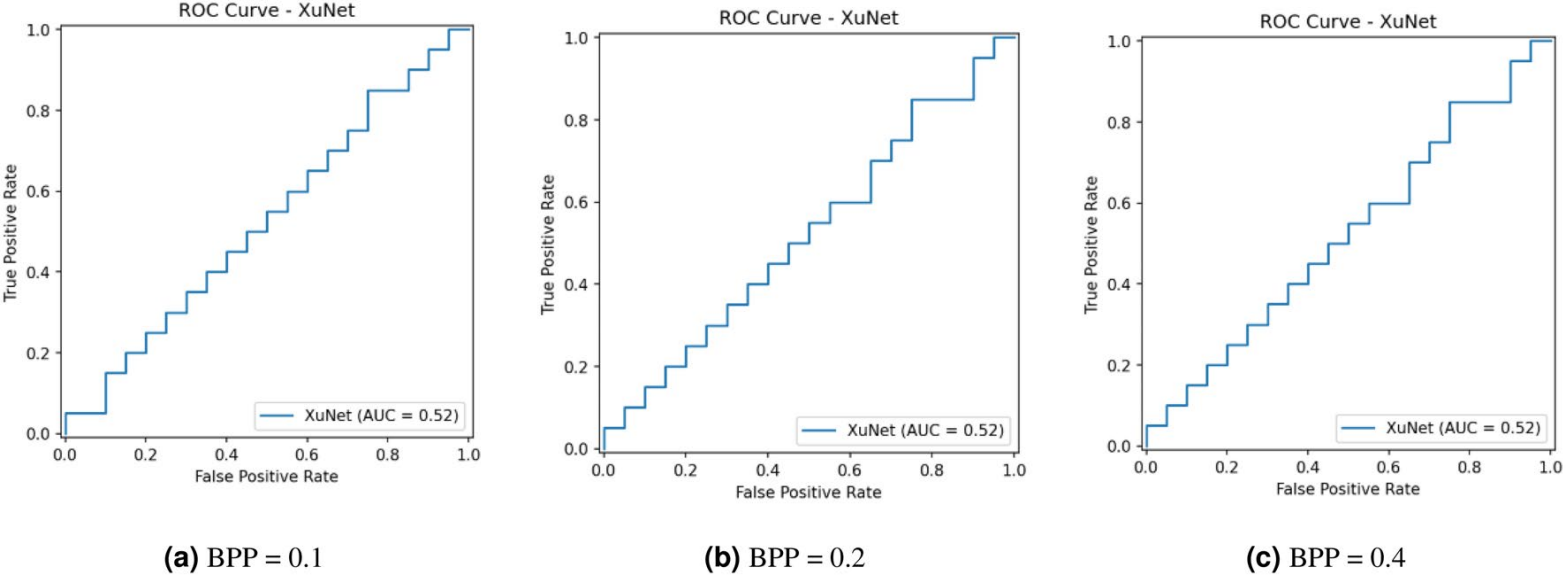
Xu-Net ROC Using ACO at different BPP.

The ROC curves in Fig. 10 show Xu-Net performance on ACO-generated stego images at three different payload rates (BPP = 0.1, 0.2, 0.4). The ROC curve maintains a constant area under the curve value of 0.52 while following the diagonal reference line throughout all panels, which indicates chance-level separability. The True Positive Rate (TPR) remains low at $\textrm{TPR}\lesssim 0.2$ when the FPR is below 0.2, which results in limited useful operating points under strict false-alarm constraints while the equal-error rate ranges between 0.49 and 0.50. The ACO embedding fails to produce artifacts that become progressively easier to learn for Xu-Net’s convolutional features at any point within this payload range because no monotonic relationship exists between BPP and learnability. The results without thresholds match the confusion matrix analysis to show Xu-Net achieves chance-level performance on this configuration without any task-specific retraining.

**Fig. 11:**
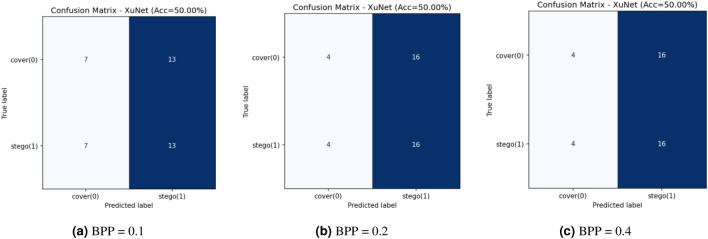
Xu-Net Confusion without ACO at different BPP.

The confusion matrices in Fig. 11 (Xu-Net without ACO) demonstrate chance-level performance at three different payload rates (BPP = 0.1, 0.2, 0.4) as the overall accuracy remains at 50%. The classifier shows consistent bias toward selecting the stego class because the right column appears dominant in every panel. The BPP = 0.1 data points [7,13] result in a sensitivity of 0.65 and a specificity of 0.35, which leads to TPR = False Positive Rate (FPR) = 0.65. The BPP = 0.2 and BPP = 0.4 data points [4,16; 4,16] produce TPR = 0.80 and True Negative Rate (TNR) = 0.20, which results in TPR = FPR = 0.80. The stego precision remains at 0.5 in all scenarios.The ROC analysis results match the observations, which show Xu-Net produces chance-level performance when it lacks ACO and task-specific training.

**Fig. 12:**
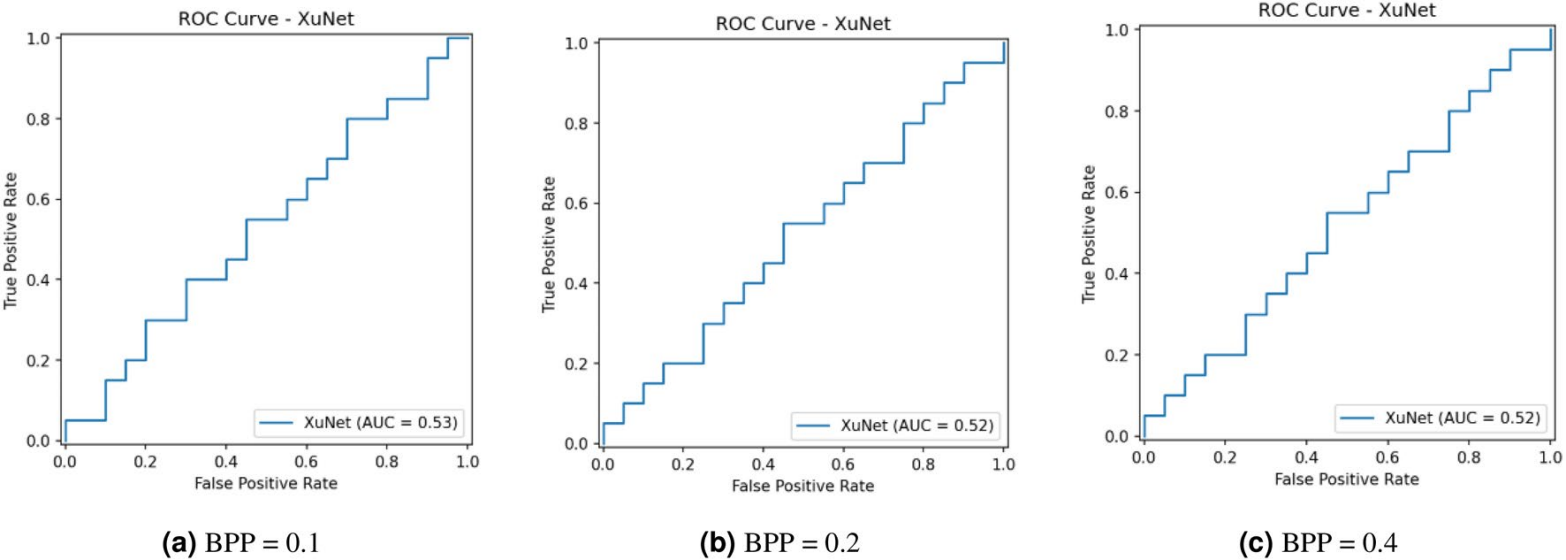
Xu-Net ROC without ACO at different BPP.

The ROC curves for Xu-Net without ACO appear in Fig. 12 at three different payload rates (BPP $=0.1, 0.2, 0.4$). The ROC curves in all cases follow the diagonal line because the AUC values remain at 0.53, 0.52, and 0.52, which shows chance-level separability. The TPR remains low at FPRs below 0.2 ($\textrm{TPR}\lesssim 0.2$), which results in limited useful operating points for the system; the system achieves equal error rates between 0.49 and 0.50 and maintains a balanced accuracy of 0. The system’s performance shows no relationship with BPP variations between 0.1 and 0.4 BPP because the features of Xu-Net remain unlearnable for ACO-free stego detection. The threshold-free results, which include confusion matrices demonstrate that Xu-Net achieves chance-level performance without task-specific retraining on this particular configuration.

### Ye-Net analysis

**Fig. 13:**
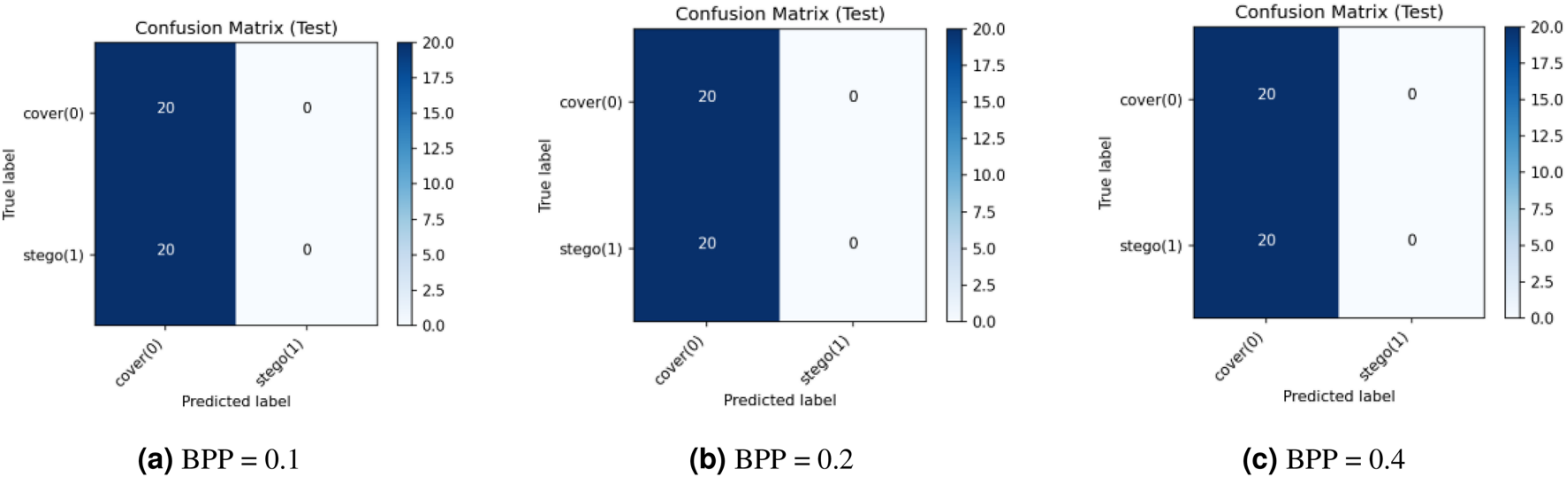
Ye-Net confusion using ACO at different BPP.

Figure 13 shows that the Ye-Net model selects the trivial ”all-cover” decision at $\textrm{BPP}=0.2$ and $0.4$ while showing the same payload-independent behavior at $0.1$. The test results with 20 cover images and 20 stego images produce $\textrm{TN}=20,\, \mathrm {False Positive (FP)}=0,\, \mathrm {False Negative (FN)}=20,\, \textrm{TP}=0$ which results in an overall accuracy of 0. The stego recall equals 0, and the cover specificity equals 1, which results in a balanced accuracy of 0.50. The stego precision becomes undefined because no samples are classified as stego, so we use 0 as the default value, which results in $F1_{stego}=0$. The precision value for the cover class equals 0.5, while the recall value equals 1, which results in an F1 score of 0.667 for the cover class and a macro-F1 score of 0.333. The Matthews correlation coefficient shows a value of 0 as the model predicts all samples as the same class. The results show that ACO-based embeddings maintain their indistinguishability from Ye-Net features across this payload range, while the detector probably needs retraining and calibration with ACO stego data. In^10^, the authors show that the Ye-Net performs even worse as a detector in this context, which is actually a positive result from the security perspective. Its accuracy fluctuates roughly between 0.45 and 0.52 across all datasets and edge operators, with several configurations dropping below 0.50, which is worse than random guessing. Such behavior indicates that the statistical traces left by the embedding are effectively hidden from Ye-Net’s feature space. Taken together, the Xu-Net and Ye-Net results show that the proposed scheme achieves a high level of resistance against modern deep steganalysis: even powerful CNN-based detectors cannot exploit consistent patterns to separate stego from cover images with confidence.

**Fig. 14:**
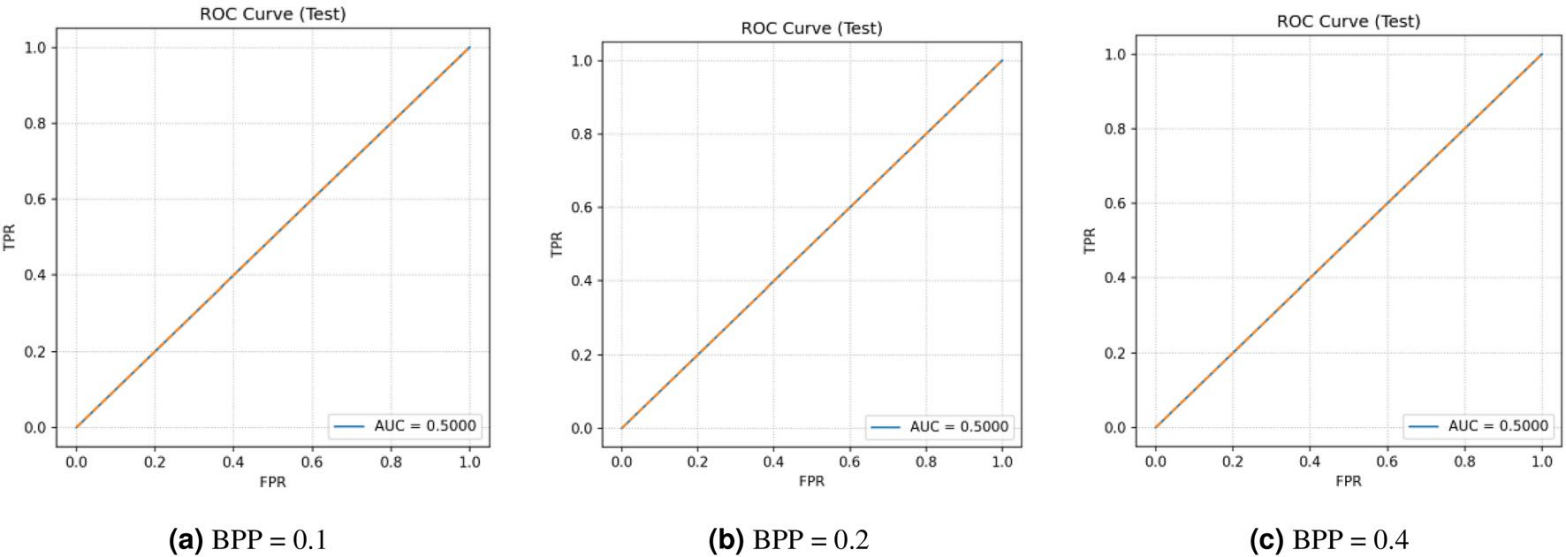
Ye-Net ROC using ACO at different BPP.

Figure 14 shows that the ROC curves of Ye-Net when tested against ACO-generated stego at $\textrm{BPP}=0.1, 0.2, 0.4$ show diagonal behavior, which results in an AUC of 0.500 because the TPR matches the FPR at every threshold, leading to an Equal Error Rate (EER) of 0.50 and balanced accuracy of 0.50. The image steganography results show random-level behavior because Ye-Net fails to detect ACO artifacts at any tested payload level, which results in no exploitable signal for the detector.

**Fig. 15:**
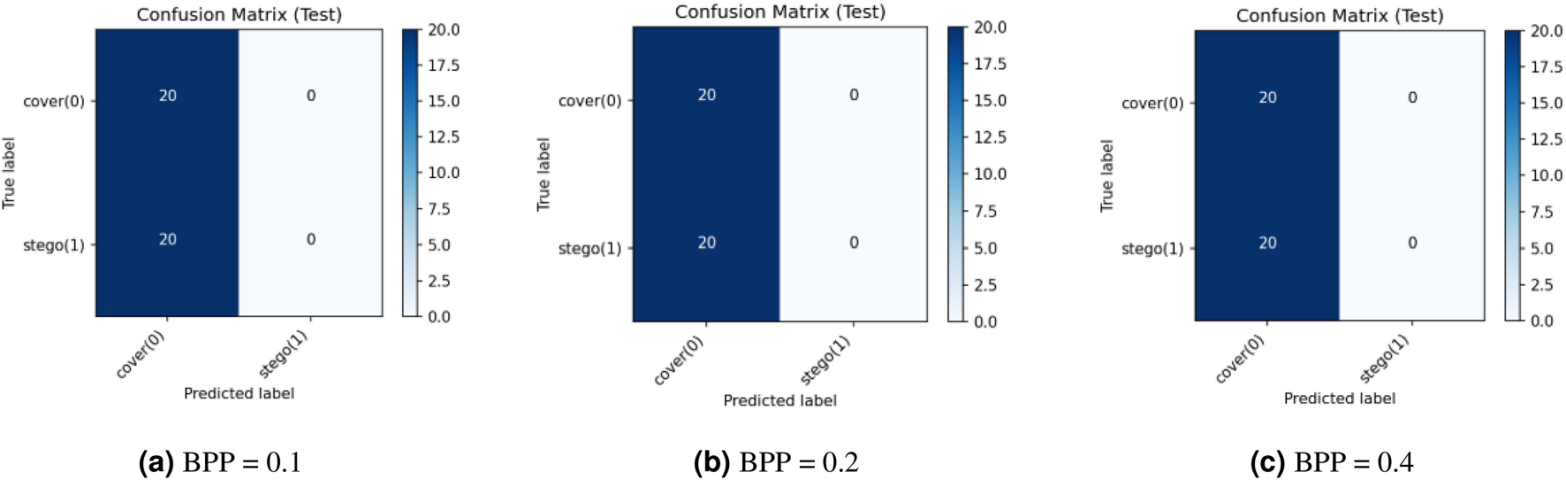
Xu-Net Confusion without ACO at different BPP.

The confusion matrices in Fig. 15 show Xu-Net makes an all-cover decision when evaluated on non-ACO stego at BPP values of 0.1, 0.2, and 0.4. The test results from a balanced dataset containing 20 cover images and 20 stego images produce TN=20 and FP=0, FN=20, and TP=0, which results in an overall accuracy of 0.50 and random classification performance. The class-wise evaluation shows that TPR equals 0 and TNR equals 1, and balanced accuracy equals 0.50 while stego precision is undefined at 0/0, so it is set to 0, which results in F1 stego=0, F1 cover=0.667, macro-F1=0.333, and Matthews Correlation Coefficient(MCC)=0. The non-ACO stego artifacts in image steganography remain undetectable to Xu-Net features at all tested payload levels, which indicates random-level behavior.

**Fig. 16:**
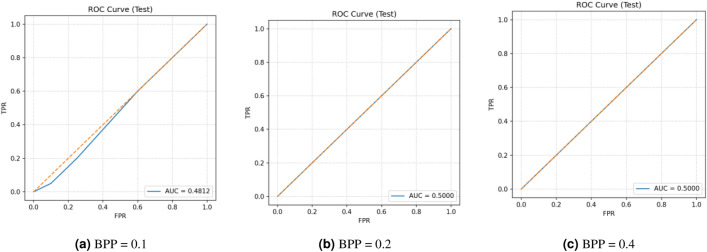
Xu-Net ROC without ACO at different BPP.

The performance of Xu-Net at three different payloads (BPP = 0.1, 0.2, 0.4) without ACO appears in Fig. 16. The ROC curves in panels (b) and (c) follow the diagonal line, which produces an AUC value of 0.500, indicating random-level separability because TPR equals FPR and EER reaches 0.50. The AUC value reaches 0.481 at BPP = 0.1, which indicates weak inverse ranking because of small-sample noise or threshold effects and non-informative discrimination. The performance of Xu-Net does not show any improvement when BPP increases from 0.1 to 0.4. The non-ACO stego appears similar to the cover image to Xu-Net at these payload values, which results in unreliable separation according to this specific detector.

### Comparing with previous work

The following table (Table 7) presents a comparison of recent steganographic methods according to their performance in terms of imperceptibility, capacity, security, and robustness. The current JPEG-domain research on BOSSbase (Break Our Steganographic System image dataset) achieves PSNR values between 35–52 dB when working with 0.01–0.5 bits per non-zero AC (bpnzAC) (non-DC frequency) coefficients in the DCT (Discrete Cosine Transform) domain) capacity and maintains security against both classical feature-based and CNN (Convolutional Neural Network) steganalyzers–namely DCTR (Discrete Cosine Transform Residual features), GFR (Gabor Filter Residual features), and SRNet (a deep residual CNN steganalyzer)–while showing robustness through error rates at JPEG quality levels (Q = quality factor 65–95).

The proposed method demonstrates superior imperceptibility through PSNR values between 55-64 dB, SSIM values between 0.9978-0.9999, IF values of 0.99, and MSE values near zero while operating at pixel-domain payloads between 0.09-0.39 BPP with a declared maximum of 104857 pixels and asserts protection against XU–Net and YU–Net. The results demonstrate a beneficial capacity-distortion relationship and comparable detection performance, but the evaluation becomes challenging because the papers use different payload measurement units (bpnzAC vs. BPP) and lack complete information about robustness. The proposed method demonstrates outstanding imperceptibility at various payload levels, yet its security performance remains promising, although a complete comparison requires standardization of payload units and complete steganalysis error/accuracy results for all detectors and JPEG/noise robustness testing under established protocols.

**Table 7:** Comparison between our work and related works based on bossbase dataset.

Paper	Dataset	Imperceptibility	Capacity	Security	Robustness
^20^	BOSSbase	PSNR>35	0.05-0.5 bpnzAC	DCTR= secure GFR= secure	Error rate from JPEG compression Q 75, Q95
^19^	BOSSbase	PSNR>50	0.01-0.1 bpnzAC	DCTR= secure GFR= secure	Error rate from JPEG compression Q 65, Q 75, Q 85
^21^	BOSSbase	PSNR>52	0.05-0.3 bpnzAC	DCTR= secure GFR= secure	Error rate from JPEG compression Q 75, Q 85, Q 90
^22^	BOSSbase	NA	0.1-0.5 bpnzAC	DCTR= secure SR net= secure	Error rate from JPEG compression Q 70
^23^	BOSSbase	NA	NA	Lowest detection accuracy in (SRM, Deng-Net, SRNet, Yedroudj-Net, Ye-Net), improving security by up to 5.39%	NA
^10^	BOSSbase	MSE= 0.0000 PSNR= up to 39.85 dB SSIM= 0.997 IF= 0.99	Max= 1,572,864 bits BPP= 8	XU-Net= 0.45–0.67=Secure YU-Net= 0.45–0.67=Secure	Robust
Proposed work	BOSSbase	MSE= 0.0000 PSNR= 55-64 SSIM= 0.9978-0.9999 IF= 0.99	Max= 104857 pixels BPP= 0.09-0.39	XU-Net= Secure YU-Net= Secure	NA

## Future work

The research needs to focus on three main areas, which include channel robustness, learning-based guidance, and scalability improvements. The system needs improvement in lossy pipeline resistance through the implementation of transform-domain embedding (DCT/DWT) and matrix or syndrome-trellis coding and channel-aware methods, which will protect image quality during compression, resizing, and filtering operations. The system requires a combination of learned cost/attention maps from GANs and reinforcement learning for distortion modeling and multi-scale attention to achieve better security against advanced detectors while maintaining side-information-free regeneration. The system requires color image, video support as well as per-region BPP adaptation, and cross-dataset and real-platform testing to validate its practical deployment capabilities.

For future research, the proposed ACO-guided content-adaptive steganographic framework can be extended to support higher embedding capacity while maintaining imperceptibility and security, particularly under real-time constraints. One promising direction is to incorporate adaptive multi-bit embedding strategies, where the payload allocation per pixel or block is dynamically adjusted based on learned saliency, texture, or distortion-cost maps rather than fixed-rate LSB substitution. Additionally, integrating learning-based guidance, such as CNN- or GAN-derived attention and cost maps–can enable more accurate identification of embedding-tolerant regions, allowing the system to safely increase payload density in complex areas while preserving visual fidelity. From a real-time perspective, lightweight neural predictors can be employed to approximate or partially replace iterative ACO optimization, significantly reducing computational overhead and enabling deployment in time-sensitive environments, such as live image transmission or video streams. Furthermore, extending the framework to multi-scale or transform-domain embedding (e.g., DWT or DCT) may provide additional degrees of freedom for capacity expansion and robustness under compression and channel distortions. These extensions would allow the proposed approach to scale toward higher-capacity, real-world steganographic applications while retaining its content-aware and security-oriented design.

## Conclusion

The research introduces a content-based LSB steganography system which combines an LSB-robust guidance map (LSB zeroing and light Gaussian smoothing and spectral-residual saliency and gradient magnitude and uniform quantization) with block-wise ACO to select indices for spatial distribution and reproducibility. The system uses RSA-derived domain-separated seeds for deterministic re-indexing, which removes side information, while hybrid cryptography (AES-GCM with RSA-OAEP and optional Hamming (7,4) on the wrapped key) provides confidentiality and authenticated recovery. The system maintains high imperceptibility through PSNR and SSIM values that approach lossless quality while providing exact rate control and complete extraction success in normal operating conditions. The system shows no better than random performance in steganalysis tests using CNN detectors (Ye-Net and Xu-Net) because ROC curves approach the diagonal line with AUC values close to 0.5 and sometimes produce all-cover decisions.

## Data Availability

The experimental code can be accessed through the following link: Experimental Code.
